# Genome‐Wide Identification, Molecular Evolution, and Expression Divergence of *CLC* , *ALMT* , *VDAC* , and *MSL* Gene Family in Barley

**DOI:** 10.1002/fsn3.70110

**Published:** 2025-03-22

**Authors:** Qingfeng Zheng, Haiyang Tang, Yuan Qin, Duo Liu, Guang Chen, Tao Tong, Ying Fu, Adeel Riaz, Fenglin Deng, Zhong‐Hua Chen, Fanrong Zeng, Wei Jiang

**Affiliations:** ^1^ MARA Key Laboratory of Sustainable Crop Production in the Middle Reaches of the Yangtze River, College of Agriculture Yangtze University Jingzhou China; ^2^ Institute of Hybrid Wheat Beijing Academy of Agriculture and Forestry Sciences Beijing China; ^3^ Central Laboratory Zhejiang Academy of Agricultural Science Hangzhou China; ^4^ School of Computer Science Yangtze University Jingzhou China; ^5^ School of Science Western Sydney University Penrith New South Wales Australia; ^6^ College of Agricultural Nanjing Agricultural University Nanjing China; ^7^ Xianghu Laboratory Hangzhou China

**Keywords:** anion channel, drought, evolution, expression analysis, gene family, *Hordeum vulgare* L., transcriptome

## Abstract

Organic and inorganic nutrients, osmotic components, associated protein cofactors, and signaling molecules regulate biotic and abiotic stresses in plants. Earlier reports suggest that plant cells activate anion channels and induce the efflux of anions at the plasma membrane under drought. Herein, *CHLORIDE CHANNEL* (*CLC*), *ALUMINUM‐ACTIVATED MALATE TRANSPORTER* (*ALMT*), *VOLTAGE‐DEPENDENT ANION CHANNEL* (*VDAC*), and MECHANOSENSITIVE CHANNEL of SMALL CONDUCTANCE‐LIKE (*MscS‐like*, *MSL*) gene family were reported in barley. Totally, 43 anion channel proteins were identified in barley at the genome‐wide level. Expression profiles of anion channel genes were obtained from public databases and verified by qRT‐PCR. In addition, the expression pattern of the anion channel gene family in multiple tissues among ten land plants showed the organs in which it is actively expressed, and 43 anion channel genes were expressed in diverse tissues, such as tillers, epidermal strips, inflorescences, and grain in barley. The expression of anion channel genes was performed in ten different cultivars and wild barley, of which 17 genes were confirmed by qRT‐PCR under drought treatment, suggesting that different cultivars have diverse anion channel genes in response to drought stress. The plants with high transcripts of these genes demonstrated stronger tolerance to drought stress and element content (e.g., potassium, calcium). The results might help to further elucidate the molecular mechanism of anion channels related to stress and provide a toolkit for enhancing the drought tolerance of barley.

## Graphical abstract

This study provides a comprehensive analysis of the *CLC*, *ALMT*, *VDAC*, and *MSL* anion channel gene families in barley. We identifies 43 anion channel proteins and examines their expression patterns in multiple tissues and drought stress. The results show that these genes play crucial roles in drought tolerance and ion homeostasis, with different cultivars exhibiting diverse responses. The findings offer insights into the molecular mechanisms of anion channels in stress adaptation and provide a foundation for developing drought‐resistant barley varieties.

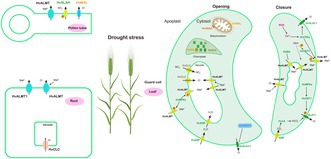

## 1 Introduction

Anion channels play important roles in plant cellular and biological functions across cell membranes, especially intracellular physiological activities and signal transduction processes (Pantoja 2021; Roelfsema et al. 2012). It has been documented that plants' growth development and their adaptive responses to stresses require the continuous trans‐membrane flow of various anions (Hedrich and Geiger 2017), which are regulated through different anion channels (Zifarelli and Pusch 2010). Each anion channel regulates one or more specific effluxes, influxes, and dynamic distributions of anions in plant cells (Saito and Uozumi 2019). Although many anion transporters have been identified and functionally verified by a series of state‐of‐the‐art biological and electrophysiological techniques (Hedrich 2012), they remain largely unknown at the molecular and evolutionary levels (Pantoja 2021).

Nitrate (${\mathrm{NO}}_{3}^{-}$), chloride (Cl^−^), and malate are categorized as the major inorganic (Feng, et al. 2020) or organic anions (Muller et al. 2017) in the plant cell. Anion proportions may vary among different species, cell types, and growth conditions but play a vital role in cellular osmosis (Hedrich 2012) and metabolism (Barbier‐Brygoo et al. 2011). For instance, ${\mathrm{NO}}_{3}^{-}$ is used for providing nitrogen, while malate is a major intermediate in osmotic regulation and carbohydrate metabolism of plant cells (Medeiros et al. 2018). Thus, the coordination of various anion channels can regulate the concentration of distinct anions in cells, thereby catalyzing common and specific functions in the plant kingdom (Barbier‐Brygoo et al. 2011).

Several anion channels, including CHLORIDE CHANNEL (CLC), ALUMINUM‐ACTIVATED MALATE TRANSPORTER (ALMT), VOLTAGE‐DEPENDENT ANION CHANNEL (VDAC), and MECHANOSENSITIVE CHANNEL of SMALL CONDUCTANCE‐LIKE (MscS‐like, MSL) were found playing vital roles in the flow and transport of anions across membranes (Hedrich 2012; Kanwar et al. 2020). The network of CLCs, ALMTs, VDACs, and MSLs plays a crucial role in coordinating plant responses to drought stress. These genes interact to regulate ion homeostasis, osmotic balance, and stress signaling pathways. ALMT transporters are rapid‐activating channels in guard cells (Dreyer et al. 2012; Roelfsema et al. 2012) and can be activated by nitric oxide (NO), abscisic acid (ABA), calcium (Ca^2+^), phosphorylation, and reactive oxygen species (ROS) (Jiang et al. 2022). The CLC family is majorly found in organellar membranes (Subba et al. 2021), and VDACs are conserved proteins in the outer membrane of mitochondria (Ashraf et al. 2021; Kanwar et al. 2022).

Drought is one of the most adverse constraints affecting plant growth and development worldwide (Kim et al. 2024; Wang et al. 2023; Zhang et al. 2024). Abiotic stress tolerance, particularly drought resilience, is a critical priority for ensuring global food security in the face of climate change (Hu et al. 2023). Plants have evolved a range of physiological traits to adapt to dehydration environments in the process of terrestrialization (Chen et al. 2017; Jiang et al. 2024). Till the present, many anion channels have been identified and proven to play important roles in plant resistance to drought stress (Pantoja 2021). For example, the overexpression of *Zea mays CLC‐d* in *Arabidopsis thaliana* (35S::*ZmCLC‐d*/*clcd‐1* and 35S::*ZmCLC‐d*) was found to enhance its tolerance to drought stress (Wang et al. 2015). The overexpression of *Medicago sativa VDAC* enhances the drought tolerance of the transgenic tobacco through regulating stress‐responsive genes (e.g., *NONSPECIFIC LIPID‐TRANSFER PROTEIN 1*, *DEHYDRIN DHN1‐LIKE,* and *HEXOKINASE‐2‐LIKE*) and osmotic homeostasis (e.g., proline contents, soluble proteins/sugars, malondialdehyde, and glutathione) (Yang et al. 2021). Overexpression of *TaVDAC1‐B* conferred high tolerance to salinity and less resistance to drought stress in *A. thaliana* (Yu et al. 2022). In *A. thaliana*, AtMSL2 and AtMSL3 also play vital roles in responding to osmotic stress by mediating the changes of volume and shape in leaf epidermal plastids (Veley et al. 2012). Compared to wild‐type plants, the *AtALMT9* mutant exhibits more sensitivity to drought stress (Qian et al. 2024). Recently, the cryo‐EM structure of *Glycine max* ALMT12 was reported and revealed the malate‐mediated activation mechanism by a domain‐twisting manner (Qin et al. 2022), which provides an essential reference for enhancing plant drought tolerance.

Barley ( *Hordeum vulgare* ) is the fourth cereal crop in the world regarding in‐field production and yield (Elakhdar et al. 2022; Xie et al. 2024). Recent advances in barley genomics, including the development of a barley pan‐transcriptome, have revealed extensive genotype‐dependent transcriptional complexity, highlighting the importance of using pangenomic resources for gene‐family characterization (Guo et al. 2025; Tong et al. 2025). These resources provide a comprehensive framework for understanding the genetic basis of stress adaptation, particularly in drought tolerance (Akbari et al. 2024). In barley, anion channels have been proved to participate in the response to diverse biotic (Koers et al. 2011) and abiotic stresses (Jiang et al. 2022). Chloride channels, such as HvCLC1 and HvCLC6 in barley, are involved in sequestering Cl^−^ into vacuoles, reducing cellular toxicity under drought stress. This mechanism helps maintain ion balance and prevents osmotic stress‐induced damage (Farooq et al. 2024). Previous studies showed that there are 10 putative *HvMSLs* in barley (Kaur et al. 2020), and the expression levels of most *HvMSLs* are particularly increased by drought and heat stresses (Jiang et al. 2022). Mechanosensitive channels, such as HvMSL1 and HvMSL3, protect cellular integrity by responding to mechanical stress caused by water deficit. They also regulate osmotic adjustments in root cells (Farooq et al. 2024). Under salinity and osmotic treatments, *Hordeum marinum* could control Cl^−^ via changing the expression of *MSLs and CLCs* in the roots and shoots (Isayenkov et al. 2020). In addition, barley mildew induced slow anion channels regulating anion efflux for stomatal closure in guard cells (Koers et al. 2011). *HvALMT8* could mediate malate efflux, which neutralizes toxic ions and regulates cellular pH. This process is crucial for maintaining root function and nutrient uptake during drought (Farooq et al. 2024). *HvALMT1* could express in mature root cells and near the lateral root junctions (Gruber et al. 2010), which takes part in anion homeostasis and stomatal function through transporting malate and other organic anions (Gruber et al. 2011; Xu, Gruber, et al. 2015) and also participates in grain germination and seed development (Xu, Gruber, et al. 2015). Voltage‐dependent anion channels in mitochondria regulate energy metabolism by controlling the flux of metabolites and ions. Under drought treatment, *HvVDAC10* in barley helps maintain mitochondrial function, ensuring energy supply for stress responses (Farooq et al. 2024). However, the functional characterization and associated features of the anion channel gene family are still unknown under drought stress in barley.

In this study, we identified the families of CLC, ALMT, VDAC, and MSL channels across the whole genome of barley and comprehensively characterized the genomic structure, domain organization, and expansion of the anion channels gene family in the plant kingdom. Furthermore, the phylogenetic relationship and gene expression profiles of these ion channel proteins were also analyzed. Besides, the plants with high expression of these genes displayed more tolerance under drought stress. Therefore, we propose that plant anion channels are one of the essential components in response to drought stress. By identifying key anion channel genes and their expression patterns, the research provides a foundation for developing drought‐resistant barley varieties, which is crucial for ensuring food security in the face of climate change.

## 2 Materials and Methods

### 2.1 Identification and Nomenclature of Anion Channel Genes in Barley

To identify the CLC (PF00654) (Liu et al. 2020), ALMT (PF11744) (Ma et al. 2020), VDAC (PF01459) (Xu et al. 2015), and MSL (PF00924) (Kaur et al. 2020) proteins in *H. vulgare* , a hidden markov model (HMM) profile of the anion channel domain (http://pfam.xfam.org/) was conducted to identify the putative anion channel proteins from genome sequences (Jayakodi et al. 2020) using the software HMMER following a previous study (Liu et al. 2020) with a cut‐off *E*‐value of < 1e^−20^. This domain was further ensured within each protein through the SMART (http://smart.embl‐heidelberg.de/) and the NCBI Conserved Domain Database (CDD) BLAST servers. Names were given to anion channel genes according to a previous study and their location on the respective chromosome (Kaur et al. 2020).

### 2.2 Multiple Sequence Alignment and Phylogenetic Relationship

Multiple alignments of sequences were conducted using MUSCLE software with the default settings. The low‐quality alignment regions and incorrect sequences with apparent splice variants were removed for improving the valid phylogeny signals (Liu et al. 2020). The phylogenetic tree was performed by MEGA 7.0 software by the neighbor‐joining method with 1000 bootstrap replicates (Chen et al. 2019). The results were displayed using iTOL 5.7 visualization (https://itol.embl.de/) (Li et al. 2022; Riaz et al. 2022).

### 2.3 Chromosomal Localization and Gene Structure Analysis

The conserved motifs and regions of anion channel proteins were predicted by the MEME tool (http://meme‐suite.org/tools/meme). The optimum motif width was ≥ 6 and ≤ 50, and the maximum number of motifs was set to 10 (Liu et al. 2020). The intron/exon distribution was determined by using the online gene structure display server program (GSDS, http://gsds.cbi.pku.edu.cn/). TBtools (version 1.082) (https://github.com/CJ‐Chen/TBtools/releases) was employed to visualize both the motif composition and gene structure (Chen et al. 2020).

### 2.4 Expression Analysis of Anion Channel Genes in Various Tissues

To create the expression profile of anion channel genes among different organs and development stages, the RNA‐seq data from various tissues in barley were retrieved from IPK (https://apex.ipk‐gatersleben.de/apex/f?p=284:49). The development stages include roots from the seedlings (10 cm shoot stage) (ROO1), shoots from the seedlings (10 cm shoot stage) (LEA), young developing inflorescences (5 mm) (INF1), developing inflorescences (1–1.5 cm) (INF2), developing tillers, 3rd internode (NOD), developing grain (5 DAP) (CAR5), developing grain (15 DAP) (CAR15), etiolated seedling, dark condition (10 DAP) (ETI), inflorescences, lemma (42 DAP) (LEM), inflorescences, lodicule (42 DAP) (LOD), epidermal strips (28 DAP) (EPI), inflorescences, rachis (35 DAP) (RAC), Roots (28 DAP) (ROO2), and senescing leaves (56 DAP) (SEN). The transcript abundance of anion channel genes was calculated according to the methods reported (Zhang et al. 2020).

Raw expression values of anion channel genes were downloaded from the CoNekT database (Proost and Mutwil 2018) for the species *Arabidopsis thaliana* , *Solanum lycopersicum* , *Oryza sativa* , *Zea mays* , *Amborella trichopoda*, *Picea abies* , *Ginkgo biloba*, *Selaginella moellendorffii*, *Physcomitrium patens*, and *Marchantia polymorpha* . Sampling conditions were categorized into root, flower, leaf, stem, female (ovaries and pistrils), seeds, male (pollen and anthers), apical meristem, and root meristem. Mean values from raw expression values per category were calculated for each gene. Pearson correlation values were clustered using affinity propagation clustering by apcluster (apcluster package, convits = 1000, maxits = 10,000, nonoise = TRUE seed = 1000) in R (v3.5.0) (Naake et al. 2021).

### 2.5 Plant Materials, Growth Conditions, and Evaluation

Two annual Tibetan wild barley genotypes XZ141 (drought tolerant) and XZ54 (drought sensitive) were used in this study (Cai et al. 2019; Wendelboe‐Nelson and Morris 2012). Uniform seeds were sterilized, germinated in the dark at 23°C for 1 day, and sown in pots with potting mixture (peat: vermiculite; 3:1). They were then grown in a well‐controlled growth room with a photoperiod of 16 h/8 h (day/night), a temperature of 23°C / 18°C (day/night), a light intensity of 225 ± 25 μmol m^−2^ s^−1^, and a relative humidity of 60%. When the plants were grown to the three‐leaf stage, barley seedlings were subjected to two water treatments: control (watering the plants as usual) and drought (stopping watering for 10 days). Both treatments were carried out with three biological replicates, and each replicate contained 3 pots. At 0 and 10 days of the treatments, barley seedlings were collected and evaluated for drought tolerance by measuring element content.

The content of elements was conducted by inductively coupled plasma mass spectrometry (ICP‐MS) according to a previous study (Feng, Liu, et al. 2020). In brief, barley shoots were oven‐dried at 65°C for 3 days, weighed, and wet‐digested with concentrated HNO_3_ using a dry thermos device (DTU2CN, Tokyo, Japan) with the following protocol: 120°C for 2 days and 150°C maintained for 1 h. The digested solution was then diluted 1:15 with Milli‐Q water, and element concentrations were measured by ICP‐MS (PerkinElmer NexION 2000, USA).

### 2.6 qPCR Analysis of Anion Channel Genes Under Drought Treatment

The 17 anion channel genes were selected for qRT‐PCR validation based on their differential expression profiles under drought stress, as identified from RNA‐seq data. Total RNA was extracted from leaves after drought condition plants by the RN38‐EASYspin Plus plant RNA extraction kit (Aidlab). The qScript cDNA Synthesis Kit (Takara) was used for cDNA synthesis, and the synthesized cDNA was then 5 times diluted for RT‐PCR. The qPCR was conducted with three biological replicates via SYBR green PCR master mix (ABI) and the LightCycler 96 Real‐Time PCR System (CFX Connect) (Pan et al. 2020; Jiang et al. 2020). Expression levels were determined in triplicate and normalized against the *HvActin* reference gene. The gene primers (designed by primer6) of qPCR are listed in Table S1. The relative expression levels of genes were measured from cycle threshold values through the $2^{-\Delta \Delta C_{t}}$ procedure (Feng, Cao, et al. 2020).

### 2.7 Statistical Analysis

Data were shown as means with standard errors of three independent biological replicates. The SPSS 26.0 software (IBM, USA) was employed to perform the analysis of variance (ANOVA) and means were compared by Duncan's multiple range tests.

## 3 Results

### 3.1 Origin and Evolution of CLC, ALMT, VDAC, and MSL in Plants

**FIGURE 1:**
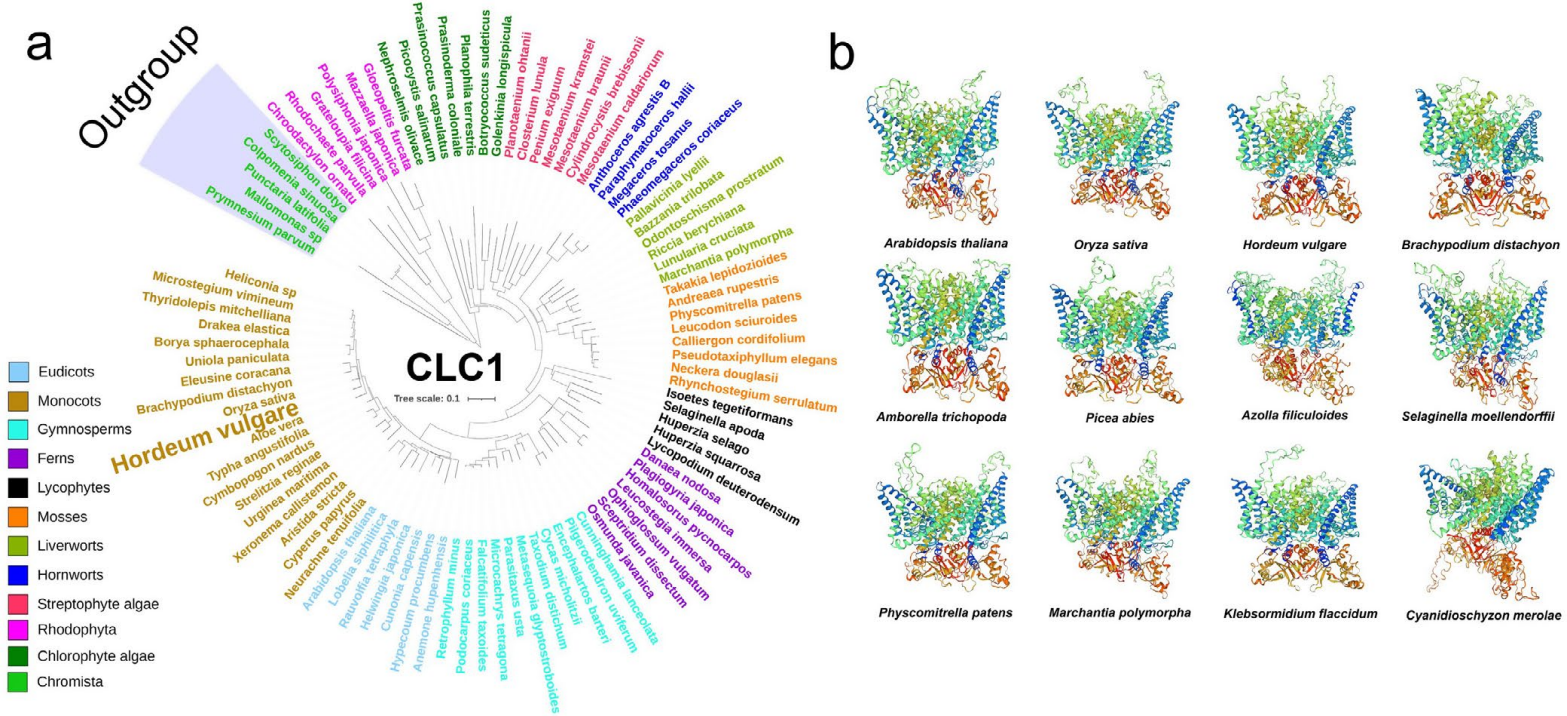
Evolution analysis of predicted CLC1 candidates in green plants (a) and predicted 3D structure of predicted CLC1 candidate representative species of the major lineage of green plants (b). All sequences were downloaded from the 1000 Plant Transcriptome and Ensembl Plants databases.

CLCs family‐related proteins are widespread among bacteria, animals, and plants (Saito and Uozumi 2019) and seem to have originated from Chromista, specifically *Prymnesium parvum* and *Colpomenia sinuosa* (Figure 1a). Protein topology prediction exhibited that CLCs are strongly conserved across representative species of angiosperms, gymnosperms, ferns, lycophytes, mosses, liverworts, and chlorophyte algae (Figure 1b).

The orthologues of ALMT1 are absent in Chromista, suggesting that they might originate from Chlorophyta *Pirula salina* and Rhodophyta *Chroodactylon ornatu* (Figure S1a). In Streptophyta, only one to three ALMT orthologues were confirmed in *Klebsormidium flaccidum* , *S. muscicola* , and *M. polymorpha* , respectively (Jiang et al. 2022). Later, the numbers were rapidly expanded to 12 in *P. abies* , 21 in *Medicago truncatula* , and 31 in *Glycine max* (Linlin et al. 2018). Taken together, ALMT channels may be diversified from a single gene in the most recent common ancestor of Streptophyta (Dreyer et al. 2012). The GmALMT12 and AtALMT1 channels are homodimers (Qin et al. 2022; Wang, Yu, et al. 2022), while the structure of other ALMTs is unclear (Figure S1b).

There are several conserved motifs including mitochondrial porin signature, β‐signal in plant VDACs (Balleza and Gomez‐Lagunas 2009). It is well conserved from lower to higher plants, which likely has originated from Chromista (e.g., *Sargassum muticum* and *Punctaria latifolia* ) (Figure S2a). Protein topology prediction displayed that VDAC1 is obviously conserved among Spermatophyta, Pteridophyta, Bryophyta, and Chlorophyta (Figure S2b).

MSLs are conserved in green plants, which likely have evolved from Chromista (e.g., *Scytosihon lomentaria* and *Mallomonas* sp.) (Figure S3a). Protein topology prediction illustrated that MSL1 is significantly conserved across representative species of vascular plants, bryophytes, and algae, which have multiple transmembrane regions (Figure S3b).

### 3.2 Expression Patterns of CLC, ALMT, VDAC, and MSL in Plants

**FIGURE 2:**
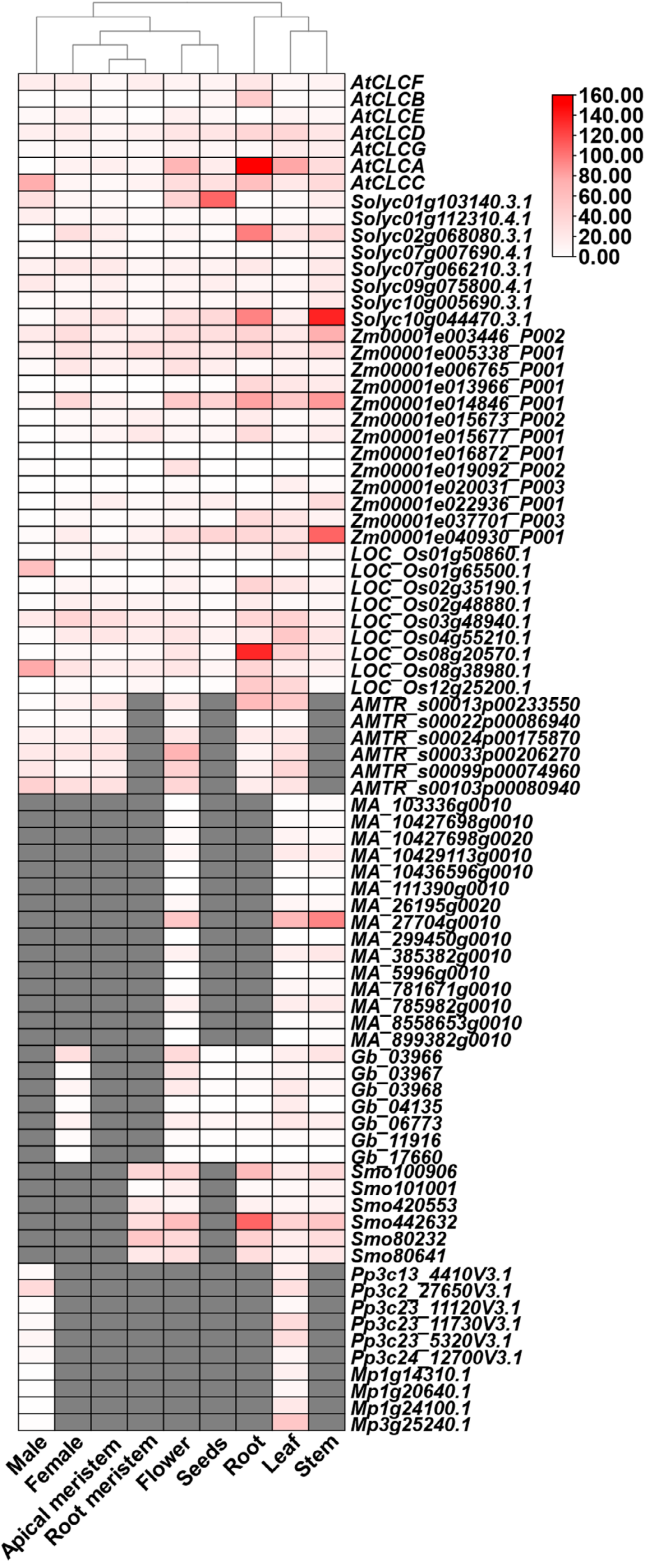
Expression patterns of *CLC* genes in plants. Expression of *CLC* genes of evolutionarily important lineages in eudicots ( *Arabidopsis thaliana* , *Solanum lycopersicum* ), monocots ( *Oryza sativa* , *Zea mays* ), Basal Angiosperm (*Amborella trichopoda*), Gymnosperms ( *Picea abies* , *Gingko biloba*), Lycophyte (*Selaginella moellendorffii*), Moss (*Physcomitrium patens*), and Liverwort ( *Marchantia polymorpha* ).

To explore the transcript level of *CLCs* in nine tissues, we compare expression profiles of the orthogroup containing *AtCLC‐A* and 81 other land plant‐specific genes (https://evorepro.sbs.ntu.edu.sg/tree/view/40922, https://evorepro.sbs.ntu.edu.sg/tree/view/42542) (Figure 2). Most *CLCs* exhibited higher expression in root and stem. In *P. abies* , *CLCs* exhibited very low expression in examined tissues except *MA_27704g0010*. A similar result was found in *G. biloba* , with only *Gb_03966* showing a higher expression level. *AtCLC‐A* was expressed specifically in root, and *LOC_Os08g20570.1*, *Smo442632*, and *Solyc02g068080.3.1* displayed the same expression pattern, indicating their equivalent function in plants. Besides, *Zm00001e040930_P001*, *MA_27704g0010*, and *Solyc10g044470.3.1* demonstrated the same expression pattern in stem. Only *Solyc01g103140.3.1* and *LOC_Os08g38980.1* showed high expression in seeds and male, respectively.

In this study, the expression pattern of 100 *ALMTs* was analyzed in root, flower, leaf, stem, female, seeds, male, apical meristem, and root meristem (https://evorepro.sbs.ntu.edu.sg/tree/view/40684) (Figure S4). In *A. thaliana* , *S. lycopersicum* , *O. sativa* , and *Z. mays* , *ALMTs* had very high expression in all examined tissues, with *AT3G18440* (*ALMT9*) and *Solyc03g096820.4.1* showing the highest values. In *M. polymorpha* and *P. patens* , however, *ALMTs* were only expressed in leaf and male. In *P. abies* , *ALMTs* were expressed in flower, leaf, and stem. These results indicate that the expression of *ALMTs* has been highly induced in monocots and eudicots during the plant evolutionary process. It was also found that some *ALMT* orthologs showed quite similar tissue expression patterns; for instance, *AT4G17585*, *Solyc06g072910.2.1*, and *LOC_Os02g49790.1* had very high expression in male, while *LOC_Os04g47930.1*, *Zm00001e041448_P001*, and *Solyc11g071350.1.1* had higher gene expression in root, indicating that these genes might have similar functions in different plant species.

The transcript levels of 61 *VDACs* were identified in root, flower, leaf, stem, female, seeds, male, apical meristem, and root meristem (https://evorepro.sbs.ntu.edu.sg/tree/view/40943) across ten land plant lineages (Figure S5). *VDACs* showed high expression in all examined tissues, especially the *AtVDAC1/3* subfamily, suggesting their crucial roles in biological processes. In *P. abies* , only *MA_157206g0010* was not expressed in the examined tissues, indicating functionally pseudogenes. In *A. thaliana* , *AT5G37610* showed the lowest expression in examined tissues. In *S. lycopersicum* , *Solyc02g067460.3.1* exhibited the highest expression in examined tissues, especially in root and root meristem. In *Z. mays* , *Zm00001e033926_P001* exhibited the highest expression in most tissues, especially in female.

*AtMSL1* and its 92 other orthologous genes were identified in CoNekT (https://evorepro.sbs.ntu.edu.sg/tree/view/42449, https://evorepro.sbs.ntu.edu.sg/tree/view/43647, https://evorepro.sbs.ntu.edu.sg/tree/view/40915). The expression pattern of *MSLs* was analyzed in nine tissues across 10 land plant lineages (Figure S6). In the *MSL1* subfamily, *Zm00001e007165_P002*, *LOC_Os02g45690.1*, and *Smo171202* displayed high expression in female, leaf, and root, respectively. In the *MSL2/3* subfamily, most *MSLs* revealed high expression in all examined tissues except *Pp3c14_8610V3.1*, especially *Smo170827* in root, flower, leaf, stem, and root meristem. In the other *MSL* subfamily, *AMTR_s00048p00206410*, *AtMSL9*, and *AtMSL4* demonstrated high expression in root meristem, flower, and root, respectively (Figure S6).

### 3.3 Identification and Phylogenetic, Conserved Domain, and Motif Analysis of CLC, ALMT, VDAC, and MSL in Barley

**FIGURE 3:**
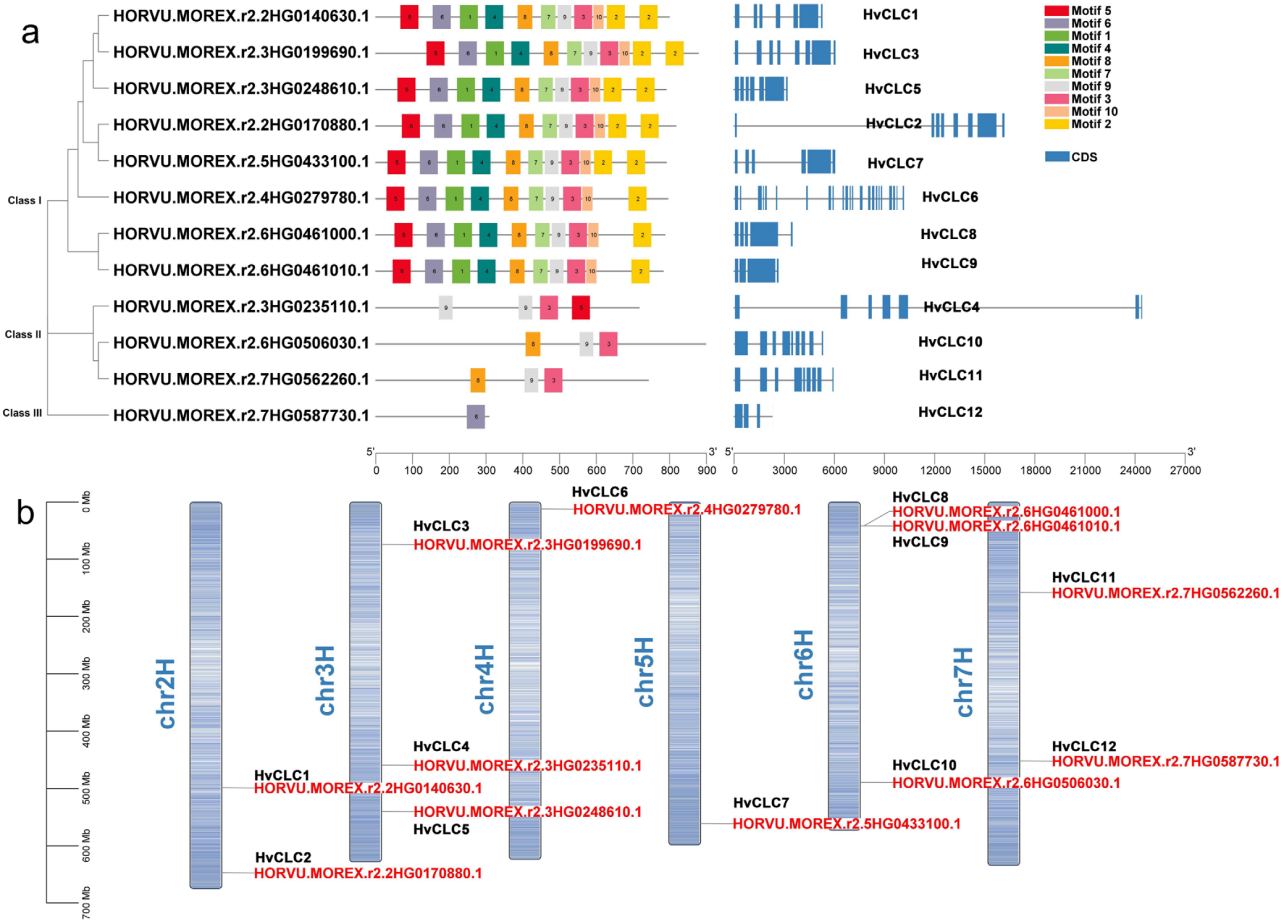
Gene structure and motif (a) and chromosomal location (b) of *HvCLCs*.

In this study, we have identified 12 CLCs (Figure 3), 10 ALMTs (Figure S7), 11 VDACs (Figure S8) and 10 MSLs (Figure S9) proteins across the whole barley genome, which was consistent with the previous studies (Jiang et al. 2022). The members of these anion channel families varied greatly in length and molecular weight. According to their exon‐intron and domain organization, all these anion channel families could be divided into three classes (Figure 3, Figures S7–S9). In the HvCLC family, the members of class I (including 2/3 members of the family) had all the 10 common motifs, while the rest of the classes only had 1–4 motifs (Figure 3a). Furthermore, most *HvCLC* genes had 3–9 exons, except *HvCLC6*, which exhibited 23 exons. In the HvALMT family, most members had 4 to 10 motifs and 2 to 7 exons, with motif‐1 and ‐2 being conserved in their gene structure. It was interesting that *HvALMT4* exhibited many fewer motifs (only one motif) but more exons (13 exons) than the other *HvALMTs* (Figure S7a). Likewise, there were 3–6 motifs contained in the HvVDAC proteins, and motif‐3, −4, and −5 were highly conserved (Figure S8a). It was also noticed that motif‐7, −8, and −9 were only identified in class III (Figure S8a), indicating that these motifs might play another crucial role in the function of the HvVDAC family. In addition, over half of the *HvVDAC* genes included 6 exons, while the amounts for the rest ranged from 9 to 11. In the HvMSL family, the members of class I and II contained 7–9 motifs, while those in class III had only one specific motif, with motif‐1 being the most conserved structure in nearly all HvMSL proteins (Figure S9a). The amounts of exons in the *HvMSL* genes varied between 4 and 14. These differences may have resulted from the absence or gain of exons during long‐term evolutionary processes.

### 3.4 Chromosomal Location and Synteny Analysis of CLCs, ALMTs, VDACs, and MSLs in Six Crops

The chromosomal location analysis revealed that *HvCLC, HvALMT, HvVDAC*, *and HvMSL* genes are widely distributed on 6, 6, 5, and 5 chromosomes in barley, respectively (Figure 3b, Figures S7–S9b). In addition, most *HvCLC* and *HvMSL* genes were located closer to the telomeric region of chromosomes, suggesting that their exchange could occur during recombination (Figure 3b, Figure S9b).

**FIGURE 4:**
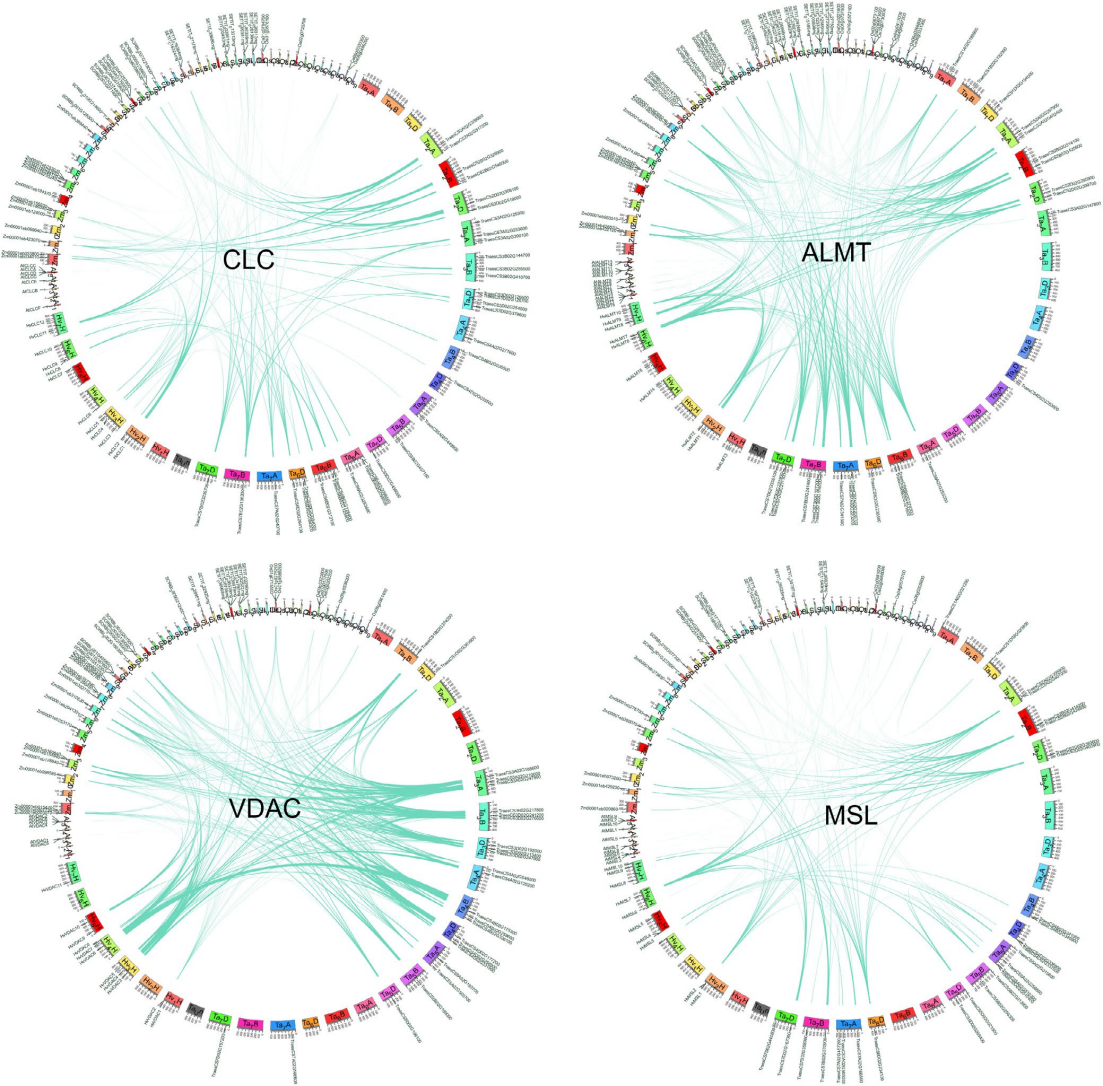
Schematic diagram of the inter‐chromosomal relationships of *CLC*, *ALMT*, *VDAC*, and *MSL* genes. Green lines indicate all syntenic blocks in the rice, wheat, barley, *Arabidopsis thaliana*, corn, sorghum, and millet genome, and black features indicate the presence of *CLC*, *ALMT*, *VDAC*, and *MSL* genes.

Duplication events play an important role in the expansion of gene families, leading to the emergence of paralogous genes during evolution (Kaur et al. 2020). They are essential for genetic variation and for acquiring additional roles of genes in order to facilitate further speciation and adaptation. We performed the analysis of gene duplication events in *A. thaliana* , *Oryza sativa* (Mao et al. 2022), *Triticum aestivum* , *Zea mays* , *Sorghum bicolor* , and *Setaria italica* to explore their contribution to the evolution of the *CLCs, ALMTs, VDACs*, *and MSLs* gene family (Figure 4). It was found that the amounts of *CLCs, ALMTs, VDACs*, *and MSLs* varied greatly between crop species, and they were unevenly distributed on different chromosomes, with some chromosomes having more genes relative to the others (Figure 4). Interestingly, the total number of *CLCs* is greater than that of *VDACs*, while *VDACs* displayed more duplication events than *CLCs* among rice, wheat, barley, *A. thaliana*, corn, sorghum, and millet. Differences in genome size and ploidy levels may account for the variation in the number of duplication events (Kaur et al. 2020). For example, a large number of duplication events occur in the complex genome of wheat (Hao et al. 2023). The collinear relationship displayed the orthologues of *CLCs, ALMTs, VDACs*, *and MSLs*, which were consistent with phylogenetic analysis.

### 3.5 HvCLCs, HvALMTs, HvVDACs, and HvMSLs Expression Patterns in Different Tissues of Barley

**FIGURE 5:**
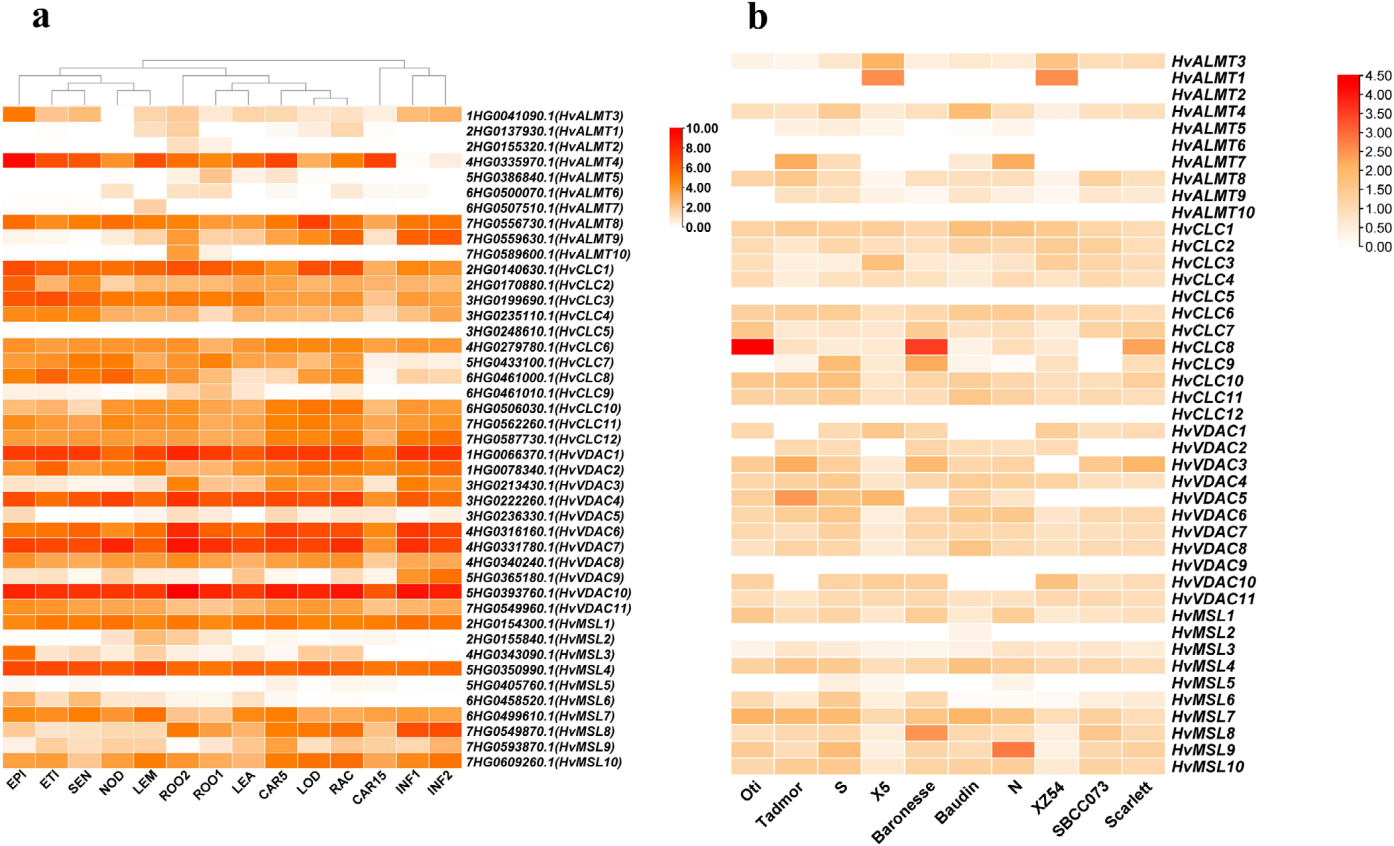
The expression of anion channel genes of organs and development stages in barley ( *Hordeum vulgare* L. cv. Morex) (a). Raw data from various tissues were retrieved from IPK (https://apex.ipk‐gatersleben.de/apex/f?p=284:49). ROO1, roots from the seedlings (10 cm shoot stage); LEA, shoots from the seedlings (10 cm shoot stage); INF1, young developing inflorescences (5 mm); INF2, developing inflorescences; NOD, developing tillers, 3rd internode; CAR5, developing grain (5 DAP); CAR15, developing grain (15 DAP); ETI, etiolated seedling, dark condition (10 DAP); LEM, inflorescences, lemma (42 DAP); LOD, inflorescences, lodicule (42 DAP); EPI, epidermal strips (28 DAP); RAC, inflorescences, rachis (35 DAP); ROO2, Roots (28 DAP); SEN, senescing leaves (56 DAP). SLAC, slow anion channel; ALMT, aluminum‐activated malate transporter; CLC, chloride channel; VDAC, voltage‐dependent anion channel; MSL, mechanosensitive channel of small conductance‐like (MscS) ‐like. The fold change of anion channel genes in different barley under drought stress (http://barleyexp.com/) (b). Drought‐tolerant (Otis, Tadmor, S, and XZ5), drought‐sensitive (Baronesse, Baudin, N, and XZ54); spanish landrace‐derived inbred line (SBCC073) and a modern cultivar (Scarlett); S and N, wild barley; X5 and XZ54, Tibetan annual wild barley.

In barley, the expression pattern of *HvCLCs, HvALMTs, HvVDACs*, *and HvMSLs* varied greatly among family members. For instance, most *HvCLCs* showed very high expression levels in different developmental tissues, whereas *HvCLC5* and *HvCLC9* were nearly absent in these tissues (Figure 5a). In *HvALMTs, HvALMT8* displayed high expression in all examined tissues, while *HvALMT2/7* were essentially not expressed in most of them. Furthermore, *HvALMT4* showed low expression in developing inflorescences but high expression in the other tissues, especially in CAR15 and EPI; *HvALMT9* exhibited specific expression in inflorescences (e.g., lodicule and rachis); and *HvALMT3* only showed high expression in epidermal strips, indicating that *HvALMTs* might play a specific role in distinct tissues (Figure 5a). In *HvVDACs*, *HvVDAC10* displayed the highest expression in nearly all examined tissues, followed by *HvVDAC1/4/6/7* (Figure 5a). However, the expression levels of *HvVDAC5/9* were much lower than those in different tissues. In *HvMSLs*, 3 *MSLs* at chr7H (*HvMSL8‐10*) showed prominent expression in reproductive tissues such as inflorescence, caryopsis, and germinating embryo (Figure 5a), suggesting that they might participate in the development of reproductive tissues (Kaur et al. 2020). In addition, *HvMSL1/4/7* showed high transcript levels in all tissues, but very low expression was observed for *HvMSL2/3/5/6/* (Figure 5a).

### 3.6 Expression Patterns of HvCLCs, HvALMTs, HvVDACs, and HvMSLs in Response to Drought Stress

The responses of *HvCLCs, HvALMTs, HvVDACs*, and *HvMSLs* genes to drought stress were examined using eight cultivated and two wild barley genotypes (Figure 5b). Our results revealed that *HvCLC1/2/3/6/7/11* were highly expressed in barley, while *HvCLC4/8/9/10* showed limited expression under drought conditions.

*HvCLC5/12* were not expressed under control or drought conditions, suggesting that they are pseudogenes. *HvCLC1* was upregulated (1.2–2.5 fold) in cultivated and wild barley under drought stress. *HvCLC6/10/11* were upregulated in cultivated and Evolution Canyon wild barley, and *HvCLC8* was highly induced (over 10‐fold) in Otis and Baronesse (Harb et al. 2020; Jiang et al. 2022). It was interesting that most *HvCLCs*, except *HvCLC1/3*, were significantly repressed in Tibetan wild barley (XZ5 and XZ54) under drought stress. The response of *HvALMTs* to drought stress differed greatly between genotypes. It was clearly observed that *HvALMT1/3* was significantly induced (2.2–5 fold‐change), while *HvALMT4/8/9* was strongly reduced (0.2–0.5 fold‐change) by drought in Tibetan wild barley (Figure 5b; Chen et al. 2018). However, they showed the opposite response to drought in the cultivated and Evolution Canyon wild barley genotypes. In addition, *HvALMT8* was downregulated in wild barley while induced in Tadmor (2 fold‐change) and SBCC073 (1.4 fold‐change) under drought condition. Likewise, the expression of *HvVDAC6/7/8* was induced in Evolution Canyon wild barley but reduced in Tibetan wild barley. *HvVDAC1/10* was significantly increased (1.6–2.3 fold‐change) in XZ5 and XZ54 under drought condition (Figure 5b). The expression of *HvMSL4/7* was induced (1.2–3.3 fold‐change) (Jiang et al. 2022), while *HvMSL3* was obviously reduced (0.2–0.7 fold‐change) in cultivar and wild barley (Figure 5b). *HvMSL2/5* was almost not expressed in control/drought stress. Most *HvMSLs* were downregulated in XZ5 and XZ54, while upregulated (except *HvMSL3/5*) in Evolution Canyon wild barley under the drought condition.

**FIGURE 6:**
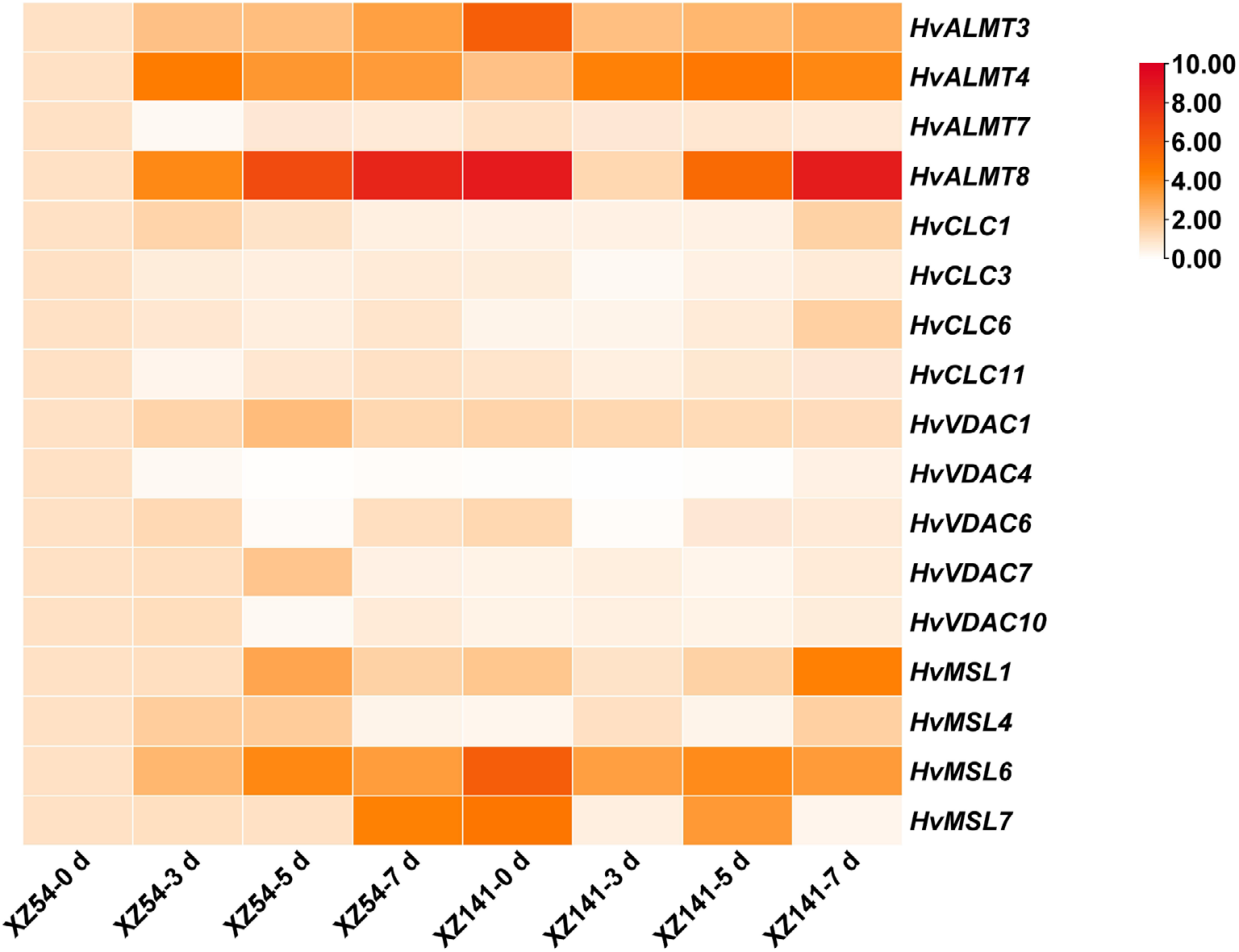
Expression analysis of anion channel genes in drought sensitive XZ54 (S) and drought tolerant XZ141 (T) barley cultivars under 0 (control), 3, 5 and 7 days of drought treatment. The value for XZ54 (control) plants was set to 1.0, and the other values of plants expressed relative to it. Differences in gene expression are indicated in color as a scale. Data are means of three independent replicates ± SD.

**FIGURE 7:**
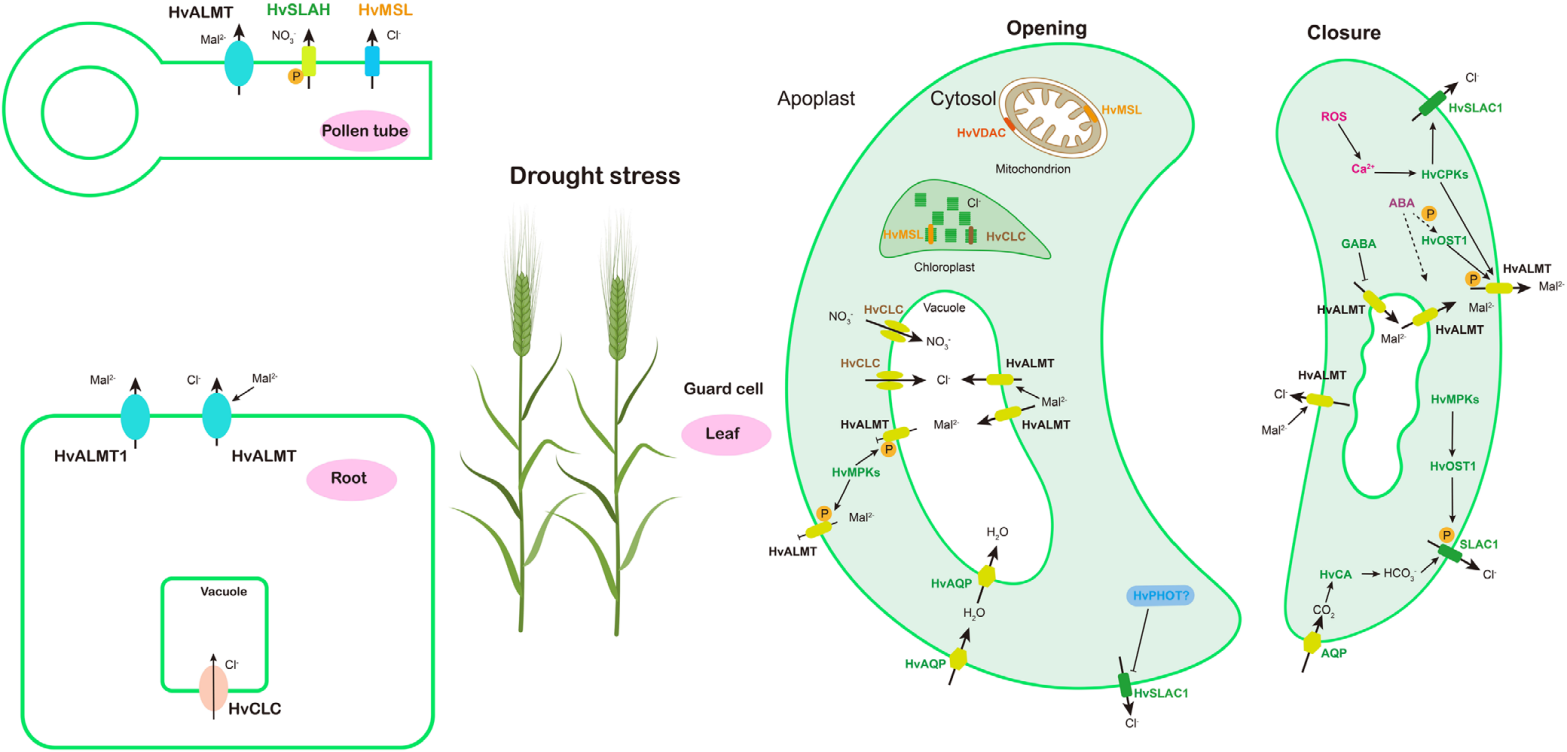
Tentative model explaining the regulatory effects of anion channel gene in barley root/leaf/pollen tube under drought stress. Thick arrow, influx/efflux of compounds; thin arrow, activation or inhibition; broken arrow, putative activation. P, stimulation by phosphorylation. Mal^2−^, malate; MPKs, Mitogen‐activated protein kinases; AQP, aquaporin; PHOT, phototropins; ROS, reactive oxygen species; CA, β‐carbonic anhydrase; CPKs, Calcium‐dependent kinases; GABA, gamma‐aminobutyric acid; SLAC, slow anion channel; ALMT, aluminum‐activated malate transporter; CLC, chloride channel; VDAC, voltage‐dependent anion channel; MSL, mechanosensitive channel of small conductance‐like (MscS‐like).

To elucidate the potential role of anion channels in barley plants' response to drought stress, their gene expression profiles under drought conditions were compared between drought‐sensitive (XZ54) and ‐tolerant (XZ141) Tibetan wild barley genotypes (Figure 6). Under control conditions, the transcripts of *HvCLC1/6* in drought‐tolerant XZ141 were lower than those in drought‐sensitive XZ54, while they were decreased in XZ54 but induced in XZ141 after 7 days of drought, indicating the potential involvement of *HvCLC1/6* in the drought stress response. In addition, no significant difference in the gene expression of *HvCLC3/11* was observed between XZ54 and XZ141. The expression of *HvVDAC1/7* was not affected by drought in XZ141, while it was obviously induced in XZ54 after 5 days of drought treatment. Compared to the control conditions, the expression level of *HvVDAC4* was increased and *HvVDAC10* was unaffected in XZ141, while they were decreased in XZ54 after drought stress (Figure 6). Under control conditions, the transcript levels of *HvALMTs* and *HvMSLs* were higher in the drought‐tolerant barley genotype XZ141 than in the drought‐sensitive genotype XZ54. Under drought stress, these gene families showed strong responses that varied between genotypes: *HvALMT3/8* and *HvMSL6/7* were significantly induced in XZ54 but exhibited little change or were repressed in XZ141. In contrast, *HvALMT4* and *HvMSL1* were significantly induced in both genotypes under long‐term drought. These results suggest that different barley genotypes may utilize distinct anion channel genes to respond to drought, which could explain the differences in drought tolerance (Figure 7).

### 3.7 Changes of Elemental Content in XZ54 and XZ141 Under Drought Treatment

**FIGURE 8:**
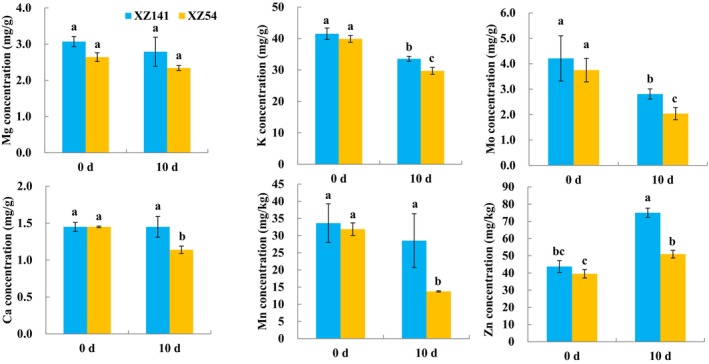
Element content of drought‐sensitive (XZ54, S) and drought‐tolerant (XZ141, T) barley seedling for drought stress. Differences in gene expression are indicated in color as a scale. Data are means of three independent replicates ± SD.

The elemental contents in XZ54 and XZ141 were measured during drought treatment (Figure 8). It was found that there is no significant difference in the contents of the examined cations between drought‐sensitive (XZ54) and ‐tolerant (XZ141) barley genotypes under the control condition. Drought stress interfered with the accumulation of these cations in barley seedlings. However, they showed much higher contents in XZ141 than in XZ54 under drought conditions. The high expression of *HvALMT8* in XZ141 is positively correlated with the high content of K^+^ and Ca^2+^ (Figures 5b and 8), which may promote cation absorption through malic acid outflow. The upregulation of Hvlclc6 in drought‐tolerant varieties (Figure 6) may reduce cytoplasmic toxicity through vacuole Cl^+^ compartmentalization and indirectly maintain K^+^ homeostasis. Our results indicated that XZ141 seedlings displayed beneficial growth because they increased the inorganic osmotic adjustment substances, which might help in plant photosynthesis and promote plant growth.

### 3.8 HvCLC, HvALMT, HvVDAC, and HvMSL Expression Profiling Under Biotic and Abiotic Stresses

**FIGURE 9:**
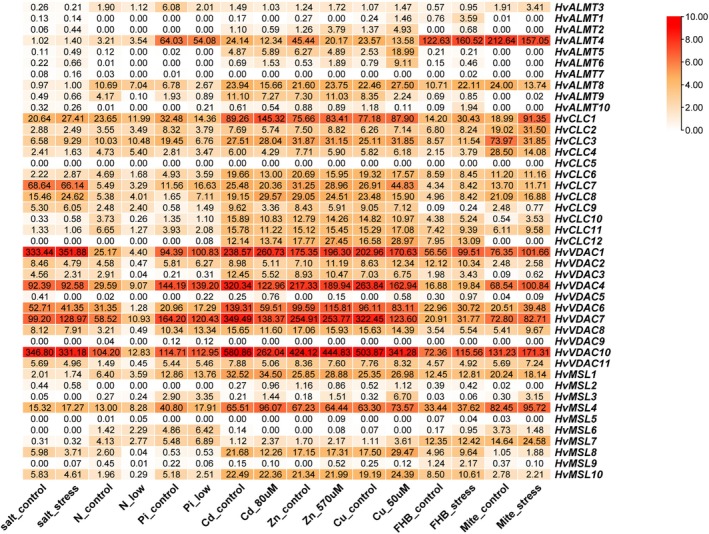
Expression analysis of *CLC*, *ALMT*, *VDAC*, and *MSL* genes in biotic and abiotic treatments.

Using the public RNA‐seq database, we analyzed the gene expression profiles of barley anion channel families in response to abiotic and biotic stresses (Figure 9). The sequences were obtained from previous studies on different environmental factors and stress treatments, including salinity (Fu et al. 2019), heavy metal (Kintlova et al. 2017), and biotic stress (Bui et al. 2018; Huang et al. 2016). *HvALMT4* was highly induced under mite and Fusarium head blight (FHB) stresses, highlighting its role in the biotic stress response. *HvALMT4/5/8/9* was significantly induced by Cu, Cd, and Zn stresses, indicating their importance in heavy metal response. Most *HvCLCs, HvVDAC1/4/6/7/10*, and *HvMSL1/4* exhibited high expression across all tested stresses, suggesting their essential roles in multiple stress responses. Conversely, *HvCLC5, HvALMT1/3/7*, *HvVDAC5/9*, and *HvMSL2/5* showed low expression in all treatments, implying negative roles or pseudo‐functionalization (Figure 9).

## 4 Discussion

### 4.1 Anion Channels Exhibit Evolutionarily Conserved Functions in Plants

The CLC/VDAC channels/transporters show higher conservation than ALMT/MSL anion channels, which can be traced to Rhodophyta (e.g., *Porphyra yezoensis* and *Cyanidioschyzon merolae*) and identified in all examined plants (Figure 1, Figure S2) (Jiang et al. 2022). The selectivity and direction of ion channels to various ions are often determined by protein topology and some key amino acid residues (Hedrich and Geiger 2017). There are 4, 5, 6, and 9 CLC orthologues in *Cyanophora paradoxa*, *Chlamydomonas reinhardtii* , moss *P. patens* , and monocot *O. sativa* , respectively (Nedelyaeva et al. 2020). The *A. thaliana* CLC proteins have higher similarity with land plants (over 48%) indicating more resistance to following the transition to land (Subba et al. 2021). In addition, the soil Cl^−^ availability and Cd uptake and its interaction with other ions play a vital role in barley Cd tolerance (Mak et al. 2019). In addition, voltage‐dependent chloride channel (VCCN) have been identified among land plants and algal species, which might be traced back to Rhodophyta *P. yezoensis* and Chlorophyta *Ostreococcus* sp. The orthologs of cation‐chloride cotransporter 1 (CCC1) were mainly confirmed in Streptophyte algae *K. flaccidum* and *S. muscicola* and land plants but not in green and red alga (Jiang et al. 2022).

Till the present, the structure of ALMT is less characterized, and it is difficult to explain their ion selectivity and relative permeability to various ions (Sharma et al. 2016). It was found that the *atalmt6* mutant shows lower malate currents in guard cell vacuoles compared to WT plants (Saito and Uozumi 2019). In addition, AtALMT12, an anion‐selective channel at the plasma membrane of guard cells, was reported to be involved in ABA‐induced stomatal closure by mediating malic acid efflux (Meyer et al. 2010). The activity of AtALMT12 was not stimulated by Al^3+^, which is distinct from its homologue AtALMT1. When responding to CO_2_, ABA, and Ca^2+^, the *atalmt12* mutant plant displayed partially impaired stomatal closure. During stomatal movements, the importance of malate has been implied before the identification of ALMTs including malate synthesis in guard cells and release from mesophyll cells (Saito and Uozumi 2019). A previous study suggests sulfate as a major element for drought‐induced stomatal closure, possibly activating AtALMT12 by sulfate (Malcheska et al. 2017).

Using six *A. thaliana* VDACs as a reference, 104 orthologs were identified from 23 representative genomes such as Rhodophyta *P. yezoensis* , Chlorophyta *C. reinhardtii* , Streptophyta *K. flaccidum* , and land plants (Jiang et al. 2022). The similarity among the members from monocots and dicots was found to be over 52%. One, two, and six VDAC orthologs were found in *C. merolae*, in *C. reinhardtii* , and, respectively, while the numbers were rapidly expanded to 11, in *Zea mays* , 15, in *Glycine max* , and 15, in *Gossypium raimondii* (Homble et al. 2012; Jiang et al. 2022). AtDTX33 and 35, members of the detoxification efflux carrier (DTX) family, were identified as an anion channel in the tonoplast of guard cells (Zhang, Zhao, et al. 2017), which exhibit vacuolar Cl^−^ influx in various types of cells. *Atdtx33/35* mutants illustrate impaired stomatal opening (Pantoja 2021).

### 4.2 Anion Channels Are Important in Plant Response to Abiotic Stress

*HvCLC‐B* (*HvCLC8*, *HORVU.MOREX.r2.6HG0461000*) was one of the candidate drought‐upregulated genes across wild barley in the African Slope of Evolution Canyon (Wang et al. 2023). *HvCLC‐C* (*MLOC_55517*) was induced in XZ5, and *HvALMT9* (*MLOC_15284* and *MLOC_57266*) was repressed in XZ5 and XZ54 under drought treatment (Chen et al. 2018). Compared to the wild type, *AtAVP1* (*vacuolar H* ^*+*^ *‐pyrophosphatase*) / *AtPP2A‐C5* (*phosphatase 2 A catalytic subunit*)/AtCLCc co‐overexpressing *A. thaliana* plants are more tolerant to drought stress through accumulating a greater amount of ions (e.g., potassium, chloride, and sodium) and upregulating more abiotic stress defense genes (e.g., *late embryogenesis‐abundant 14/4*, *Desiccation‐responsive gene 29A/B*) (Balasubramaniam et al. 2022). *ZmCLC‐d* displays upregulation under drought stress and ABA treatment (Wang et al. 2015), and *OsCLC1* can enhance drought tolerance, resulting in increased grain yield (Um et al. 2018). In *A. thaliana* , the overexpression of *GmCLCnt* improves transgenic plants salt tolerance, which is induced by cold stress as well (Zhou and Qiu 2010). The CLC family is not only involved in drought response, but also its subcellular dynamics (such as transport from Golgi apparatus to plasma membrane) have been proved to play a key role in salt stress adaptation (Rajappa et al. 2024), which suggests that barley *HvCLCs* may cooperate to cope with multiple stresses through similar mechanisms.

ALMTs are widely distributed in multiple plant tissues, regulating diverse biological functions, including aluminum (Al) resistance, symbiotic nitrogen fixation, stomatal regulation (Chen et al. 2024), mineral nutrition, and anion homeostasis (Medeiros et al. 2018). Each ALMT subunit harbors six transmembrane regions, which is typical of an anion channel because of their structure and function (Wang, Yu, et al. 2022). However, the functional characterization of ALMTs is still unknown under drought stress in barley. Anion channels (e.g., ALMT12) are the master switches of drought stress responses (Jiang et al. 2022; Roelfsema et al. 2012), which might coordinate in roots, guard cells, and pollen tubes (Gutermuth et al. 2018) in barley (Figure 5a). In addition, xylem‐derived sulfate might induce stomatal closure by ALMT12 and the ABA synthesis of guard cells under drought stress (Malcheska et al. 2017). In *Guzmania monostachia* , ammonium counteracts the adverse impacts of drought and intensifies CAM photosynthesis by enhancing malate transport (Pereira et al. 2018). The functional diversity of the ALMT family is further reflected in that *ALMT9* regulates vacuole malic acid accumulation through alternative splicing in apple (Li et al. 2024), while *AtALMT5* mediates vacuole input of fumaric acid (Doireau et al. 2024). These findings support that barley *HvALMTs* may participate in stress response by regulating the dynamic balance of organic acids.

VDACs are located in the outer membrane of mitochondria for the transport of solutes and energy and are involved in programmed cell death (Shoshan‐Barmatz et al. 2010). Overexpression of *AtVDAC2* has been reported to confer drought tolerance through an ABA‐dependent manner (Yan et al. 2009), while overexpression of *TaVDAC1* relieves tolerance to drought stress in transgenic *A. thaliana* (Guo et al. 2019). CLC proteins in mitochondria could co‐operate with VDAC to confer cold tolerance in *Z. mays* (Tampieri et al. 2011). Furthermore, overexpression of *TaVDAC1* and *ZmCLC‐d* in *A. thaliana* induces the tolerance of transgenic plants to cold stress (Guo et al. 2019; Wang et al. 2015). Under flooding stress, Al_2_O_3_ nanoparticles affect VDAC proteins by mediating membrane permeability (Mustafa and Komatsu 2016). Besides, waterlogging stress induces the expression of *TaVDAC* in wheat (Qi et al. 2018). In beetroots ( *Beta vulgaris* ), the abundance of *VDAC* was increased under flooding stress (Rojas‐Méndez et al. 2021). MSLs are a large and highly conserved family of transmembrane proteins, which exist in all life forms, including archaea, bacteria, and eukaryotes (Li et al. 2020). MSLs have been documented to respond to signals and be related to developmental processes such as cell wall damage, plant‐pathogen interactions, lateral root emergence, and pollen tube growth (Kaur et al. 2020). For instance, the significance of MSL1 has been confirmed in *atmsl1‐1* mutants under high temperature and cadmium stress (Lee et al. 2016). During normal growth and development, AtMSL1/2/3 is necessary for the maintenance of plastid osmotic homeostasis (Lee et al. 2019).

### 4.3 Anion Channels Play Important Roles in Alleviating Biotic Stress in Plants

Anion channels are also involved in basal resistance or innate immunity (Roelfsema et al. 2012). Fungal microbe‐associated molecular pattern (MAMP) chitosan induce the activation of S‐type anion channels in guard cells of barley (Koers et al. 2011). Likewise, infection with barley powdery mildew triggers anion channels and prevents light‐induced hyperpolarization at the plasma membrane, explaining why powdery mildew invading barley leaves inhibits stomatal opening (Roelfsema et al. 2012). The *atclc‐d* mutants were more resistant to the bacterial pathogen *Pseudomonas syringae* pv. tomato DC3000, and AtCLCd‐overexpressing lines showed increased susceptibility (Guo et al. 2014).

VDACs play a crucial role in plant immunity (Wang, Xu, et al. 2022). Overexpression of *Vitis piasezkii* VDAC3 in *A. thaliana* resulted in increased resistance to pathogens by preventing VpVDAC3 protein accumulation through protein post‐transcriptional regulation (Xu et al. 2021). Ubiquitin modification of TaVDAC1 in wheat was utilized by pathogenic effector protein Pst11215 to inhibit the immune response (Pan et al. 2024), suggesting that barley *HvVDACs* may play a dual role through mitochondrial function regulation in biological stress. AtMSL10 is potentially involved in the defense against the bacterial pathogen *Pseudomonas syringae* in *A. thaliana* (Basu et al. 2022). MSL10 was required for proper wound (e.g., insect attack) induced electrical and Ca^2+^ signaling by cooperating with glutamate receptor‐like proteins (Moe‐Lange et al. 2021). Besides, AtMSL4 participated in pathogen‐triggered immunity by interacting with accelerated cell death 6 (ACD6) (Basu and Haswell 2017; Zhang, Tateda, et al. 2017).

While this study provides a comprehensive genome‐wide analysis of anion channel genes in barley and their drought‐responsive expression patterns, their specific functions still need to be further verified using biotechnological approaches to elucidate the precise roles of these anion channels in abiotic stress responses. These steps will bridge the gap between transcriptional correlations and mechanistic understanding, ultimately accelerating the development of drought‐resilient barley varieties.

## Author Contributions

**Qingfeng Zheng:** formal analysis (lead), visualization (equal), writing – original draft (lead), writing – review and editing (lead). **Haiyang Tang:** writing – original draft (lead), writing – review and editing (lead). **Yuan Qin:** formal analysis (equal), writing – review and editing (lead). **Duo Liu:** writing – review and editing (supporting). **Guang Chen:** writing – original draft (supporting). **Tao Tong:** writing – original draft (supporting). **Ying Fu:** formal analysis (supporting). **Adeel Riaz:** formal analysis (supporting). **Fenglin Deng:** funding acquisition (supporting), writing – review and editing (supporting). **Zhong‐Hua Chen:** writing – review and editing (supporting). **Fanrong Zeng:** conceptualization (equal), formal analysis (supporting), funding acquisition (supporting), writing – review and editing (supporting). **Wei Jiang:** conceptualization (equal), formal analysis (lead), funding acquisition (lead), visualization (lead), writing – original draft (lead), writing – review and editing (lead).

## Conflicts of Interest

The authors declare no conflicts of interest.

## Supporting information


**Figure S1.** Phylogenetic tree analysis of ALMT1 and predicted 3D structure of ALMT proteins in *Arabidopsis*. All sequences were downloaded from the 1000 Plant Transcriptome and EnsemblPlants databases.
**Figure S2.** Phylogenetic tree analysis of VDAC1 and predicted 3D structure of VDAC1 proteins in plants and algae. All sequences were downloaded from the 1000 Plant Transcriptome and EnsemblPlants databases.
**Figure S3.** Phylogenetic tree analysis of MSL1 and predicted 3D structure of MSL1 proteins in plants and algae. All sequences were downloaded from the 1000 Plant Transcriptome and EnsemblPlants databases.
**Figure S4.** Phylogenetic analysis and expression patterns of *ALMT* genes in green plants.
**Figure S5.** Phylogenetic analysis and expression patterns of *VDAC* genes in green plants.
**Figure S6.** Phylogenetic analysis and expression patterns of *MSL* genes in green plants.
**Figure S7.** Gene structure and motif (a) and chromosomal location (b) of *HvALMTs*.
**Figure S8.** Gene structure and motif (a) and chromosomal location (b) of *HvVDACs*.
**Figure S9.** Gene structure and motif (a) and chromosomal location (b) of *HvMSLs*.

**Table S1.** List of primer sequences used in this study.

## Data Availability

All data supporting the findings of this study are available within the paper and within its Supporting Information published online.
