# Supplementary material for: Genome‐Wide Identification, Molecular Evolution, and Expression Divergence of CLC , ALMT , VDAC , and MSL Gene Family in Barley
Source: Food Sci Nutr. 2025 Mar 22;13(3):e70110. doi: 10.1002/fsn3.70110 (PMC11928749; doi:10.1002/fsn3.70110)
Supplement: Supplementary file 1 — Figure S1. Phylogenetic tree analysis of ALMT1 and predicted 3D structure of ALMT proteins in Arabidopsis. All sequences were downloaded from the 1000 Plant Transcriptome and EnsemblPlants databases. Figure S2. Phylogenetic tree analysis of VDAC1 and predicted 3D structure of VDAC1 proteins in plants and algae. All sequences were downloaded from the 1000 Plant Transcriptome and EnsemblPlants databases. Figure S3. Phylogenetic tree analysis of MSL1 and predicted 3D structure of MSL1 proteins in plants and algae. All sequences were downloaded from the 1000 Plant Transcriptome and EnsemblPlants databases. Figure S4. Phylogenetic analysis and expression patterns of ALMT genes in green plants. Figure S5. Phylogenetic analysis and expression patterns of VDAC genes in green plants. Figure S6. Phylogenetic analysis and expression patterns of MSL genes in green plants. Figure S7. Gene structure and motif (a) and chromosomal location (b) of HvALMTs. Figure S8. Gene structure and motif (a) and chromosomal location (b) of HvVDACs. Figure S9. Gene structure and motif (a) and chromosomal location (b) of HvMSLs. [file FSN3-13-e70110-s001.pptx]

## Slide 1
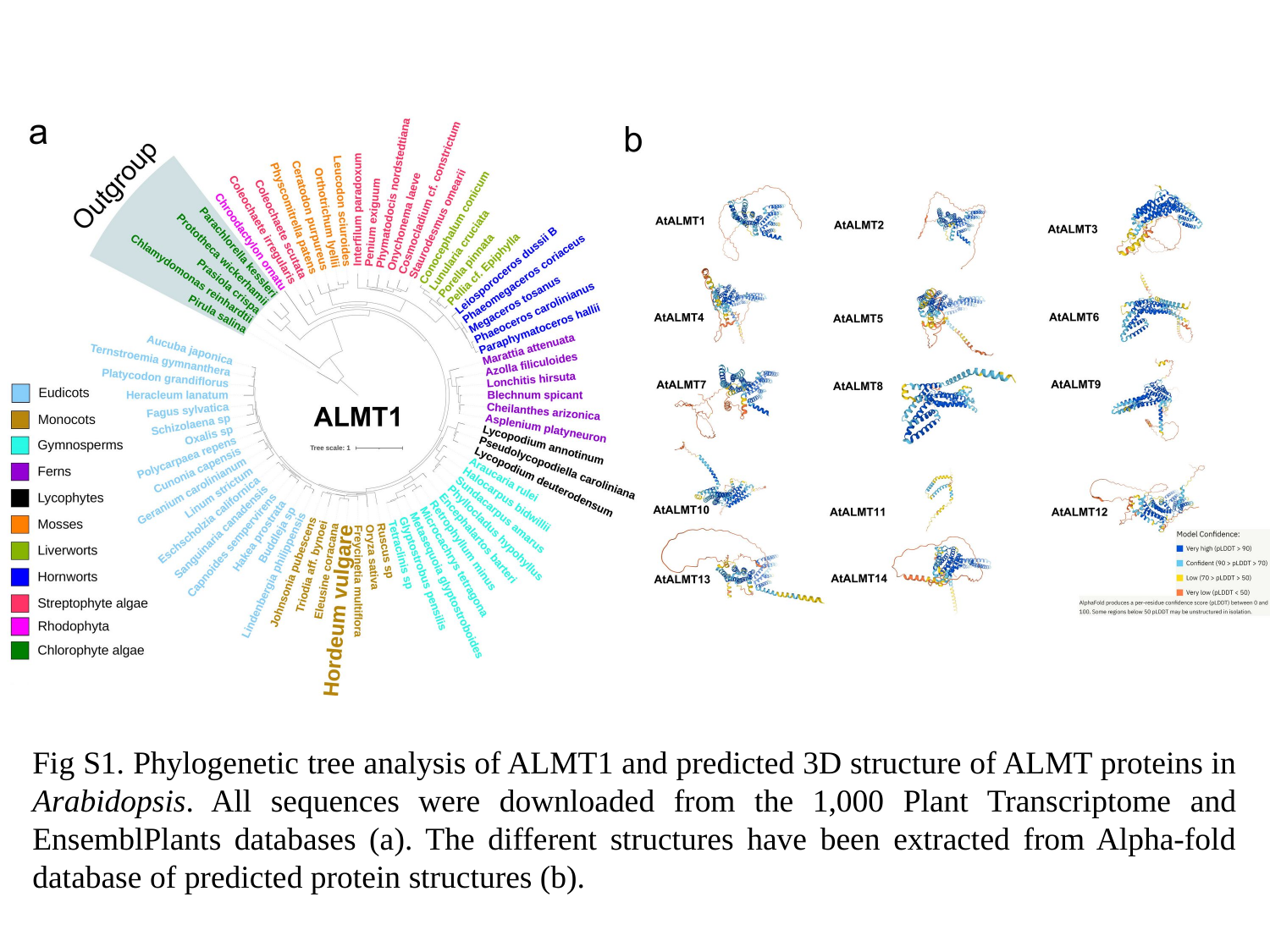

Fig S1. Phylogenetic tree analysis of ALMT1 and predicted 3D structure of ALMT proteins in Arabidopsis. All sequences were downloaded from the 1,000 Plant Transcriptome and EnsemblPlants databases (a). The different structures have been extracted from Alpha-fold database of predicted protein structures (b).

## Slide 2
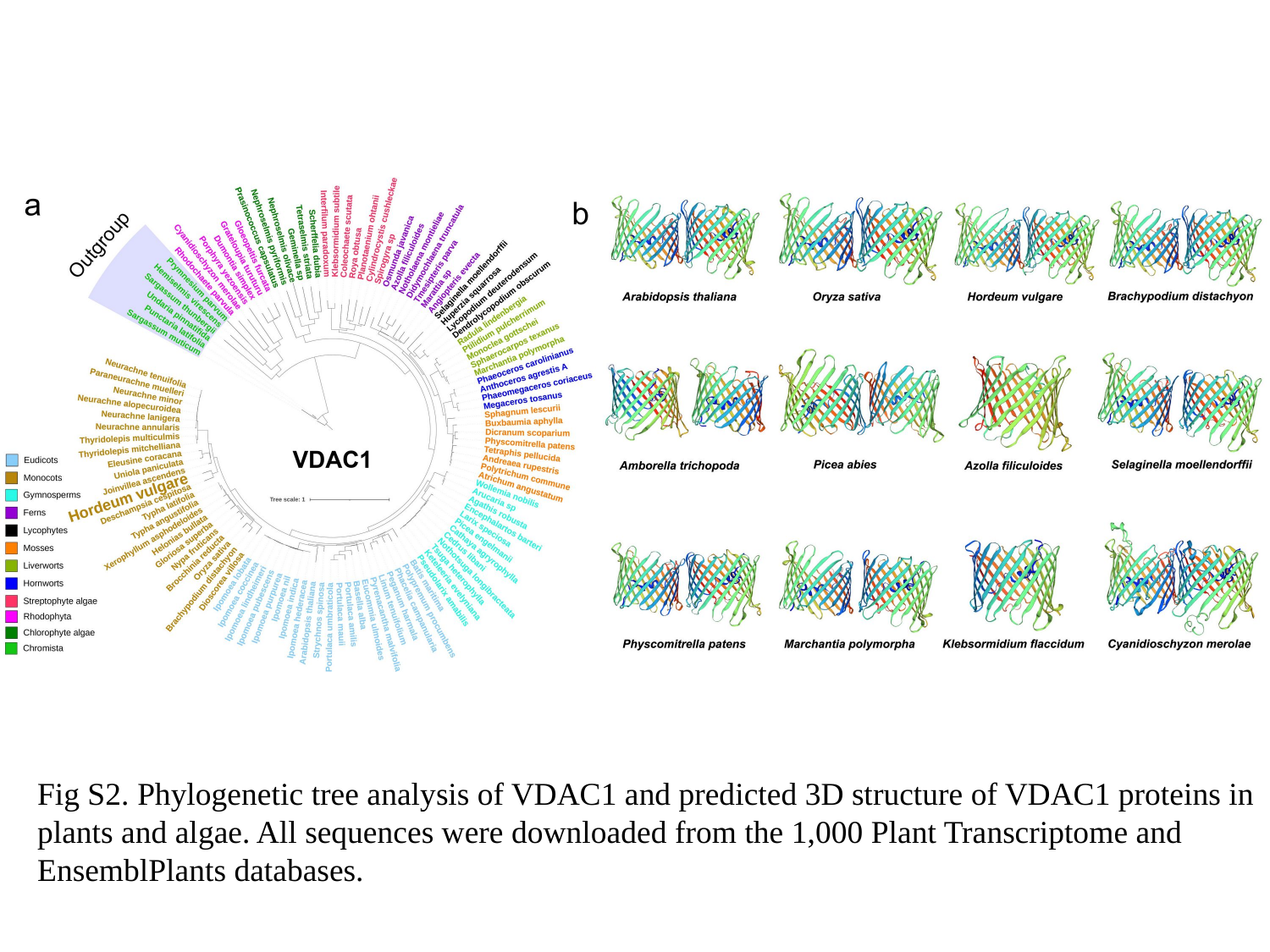

Fig S2. Phylogenetic tree analysis of VDAC1 and predicted 3D structure of VDAC1 proteins in plants and algae. All sequences were downloaded from the 1,000 Plant Transcriptome and EnsemblPlants databases.

## Slide 3
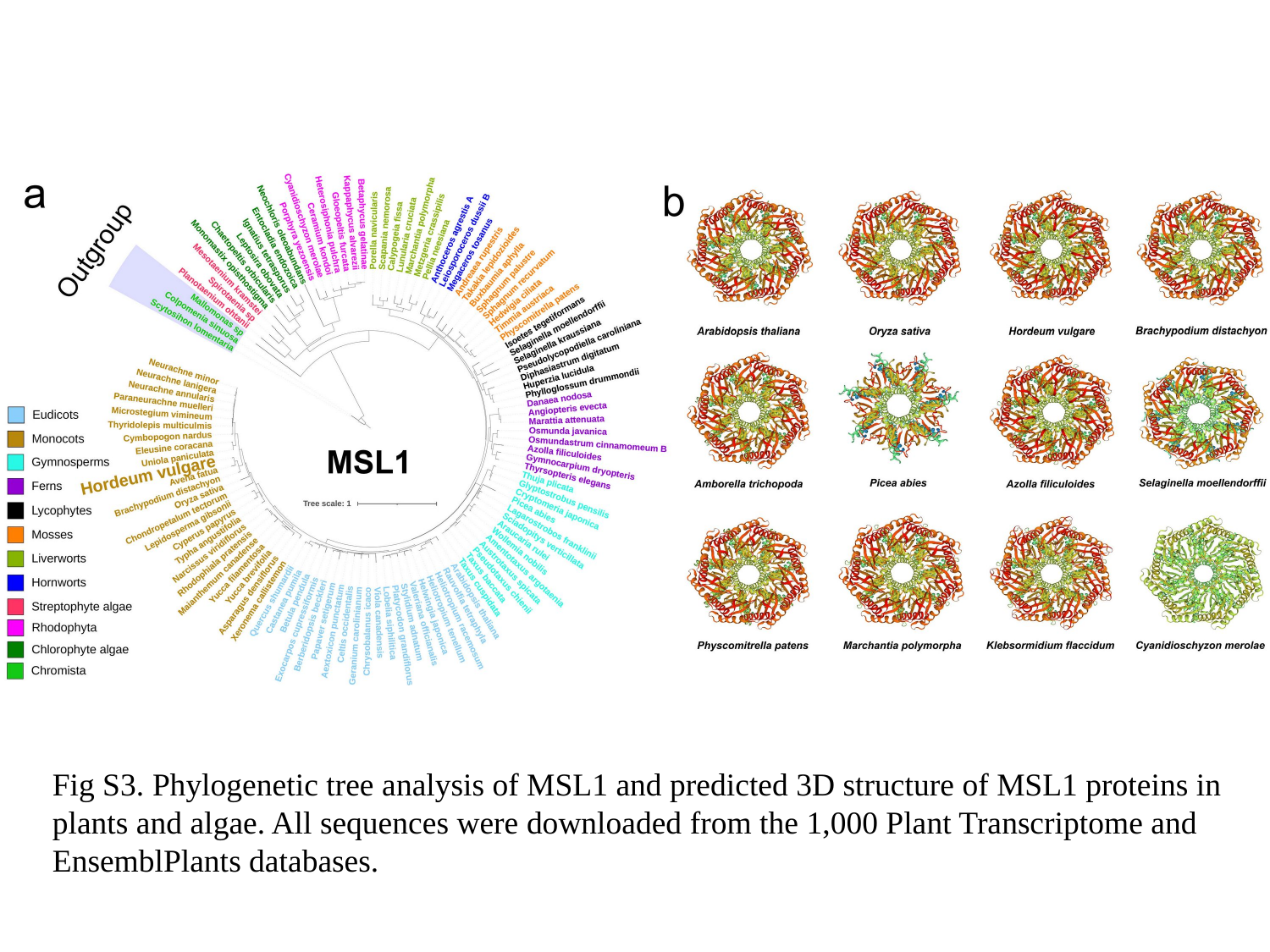

Fig S3. Phylogenetic tree analysis of MSL1 and predicted 3D structure of MSL1 proteins in plants and algae. All sequences were downloaded from the 1,000 Plant Transcriptome and EnsemblPlants databases.

## Slide 4
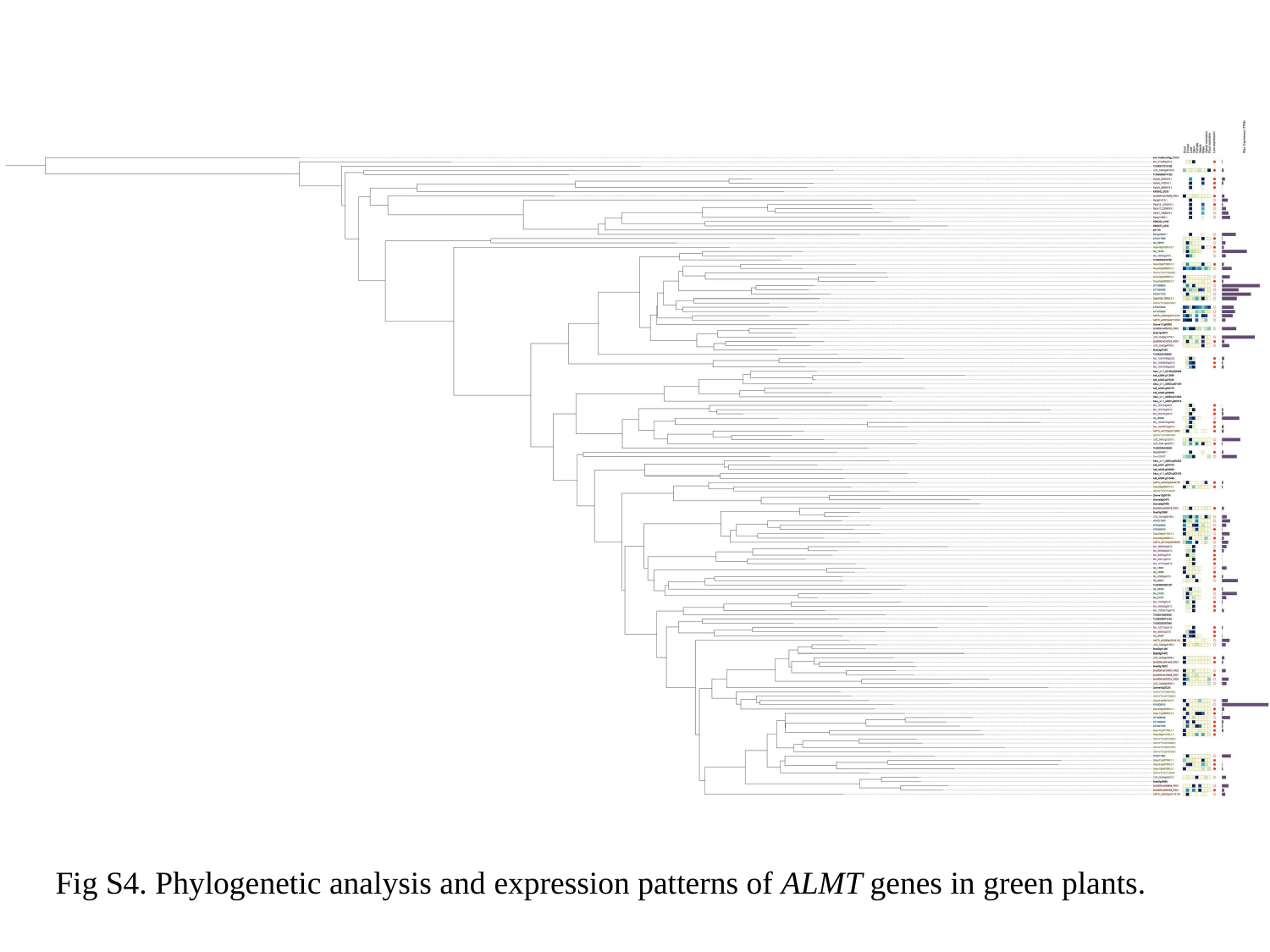

Fig S4. Phylogenetic analysis and expression patterns of ALMT genes in green plants.

## Slide 5
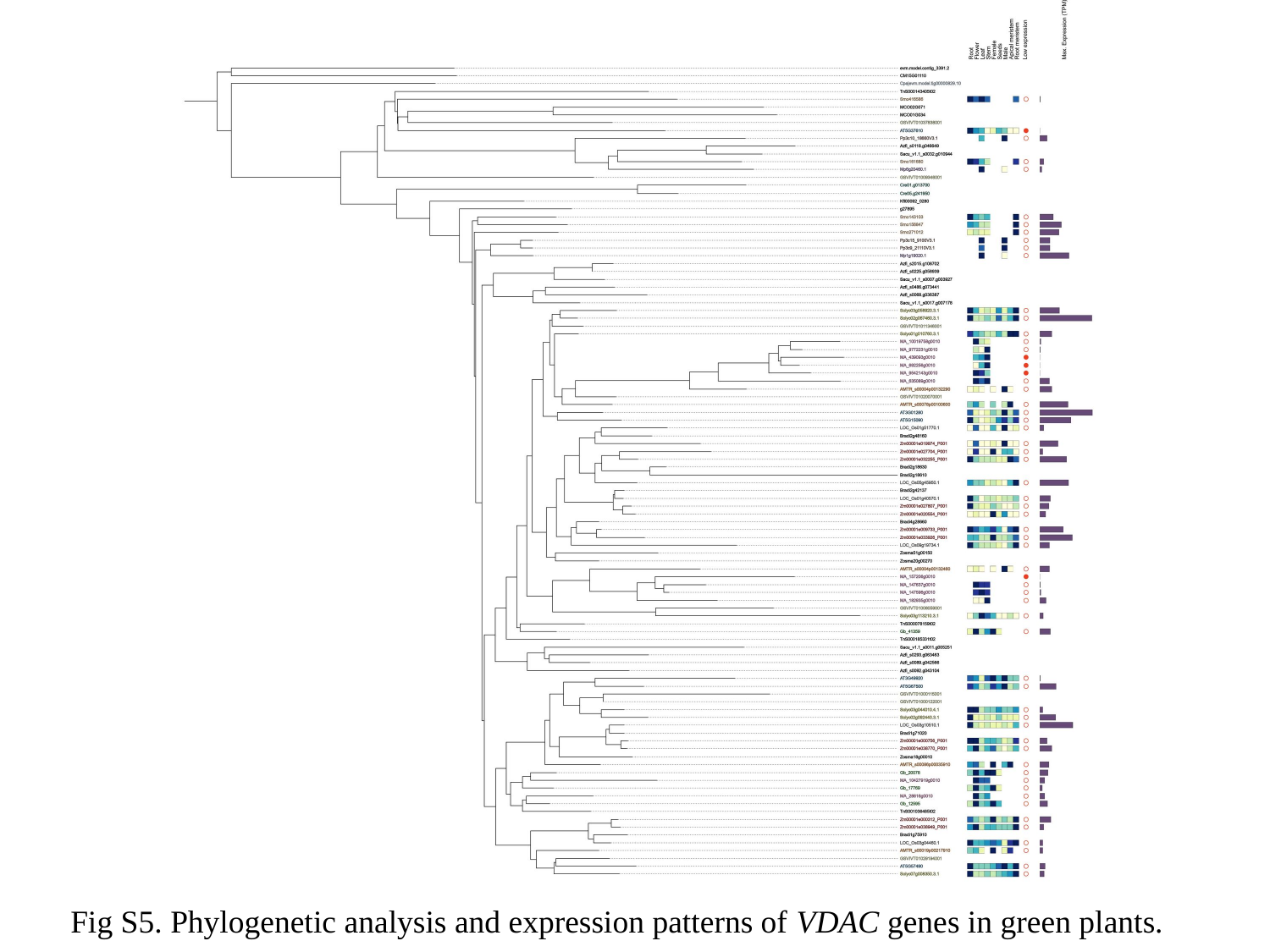

Fig S5. Phylogenetic analysis and expression patterns of VDAC genes in green plants.

## Slide 6
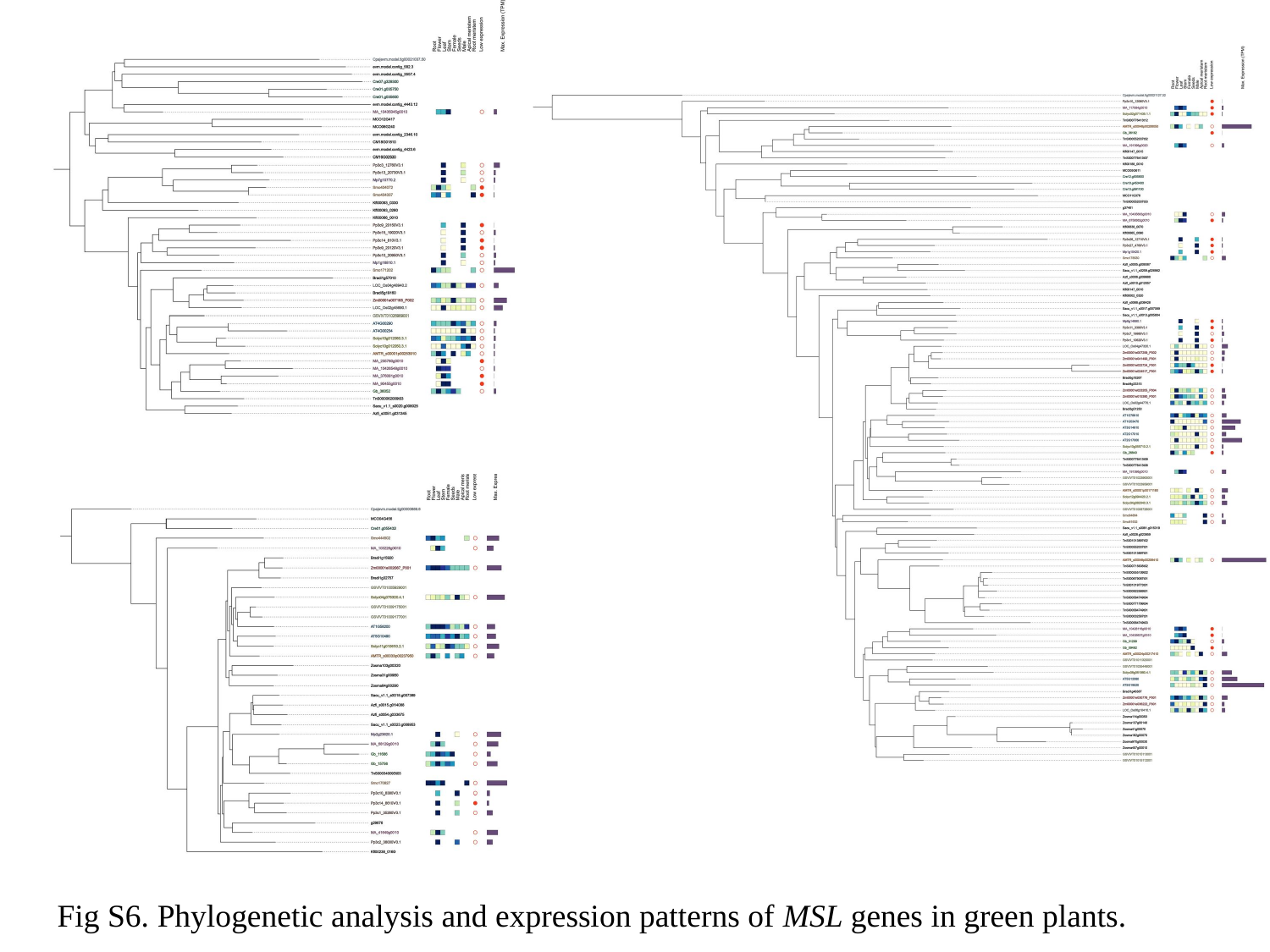

Fig S6. Phylogenetic analysis and expression patterns of MSL genes in green plants.

## Slide 7
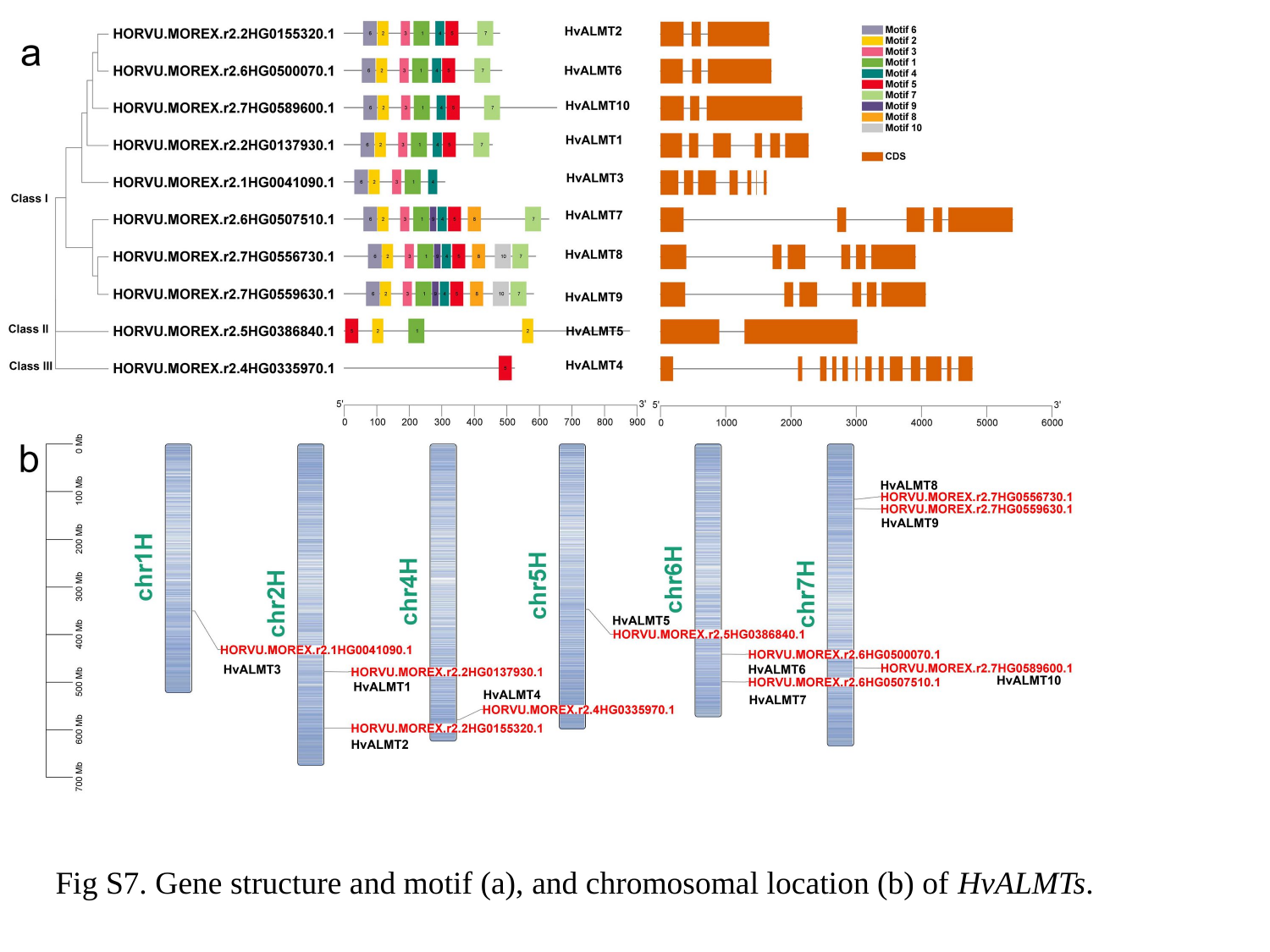

Fig S7. Gene structure and motif (a), and chromosomal location (b) of HvALMTs.

## Slide 8
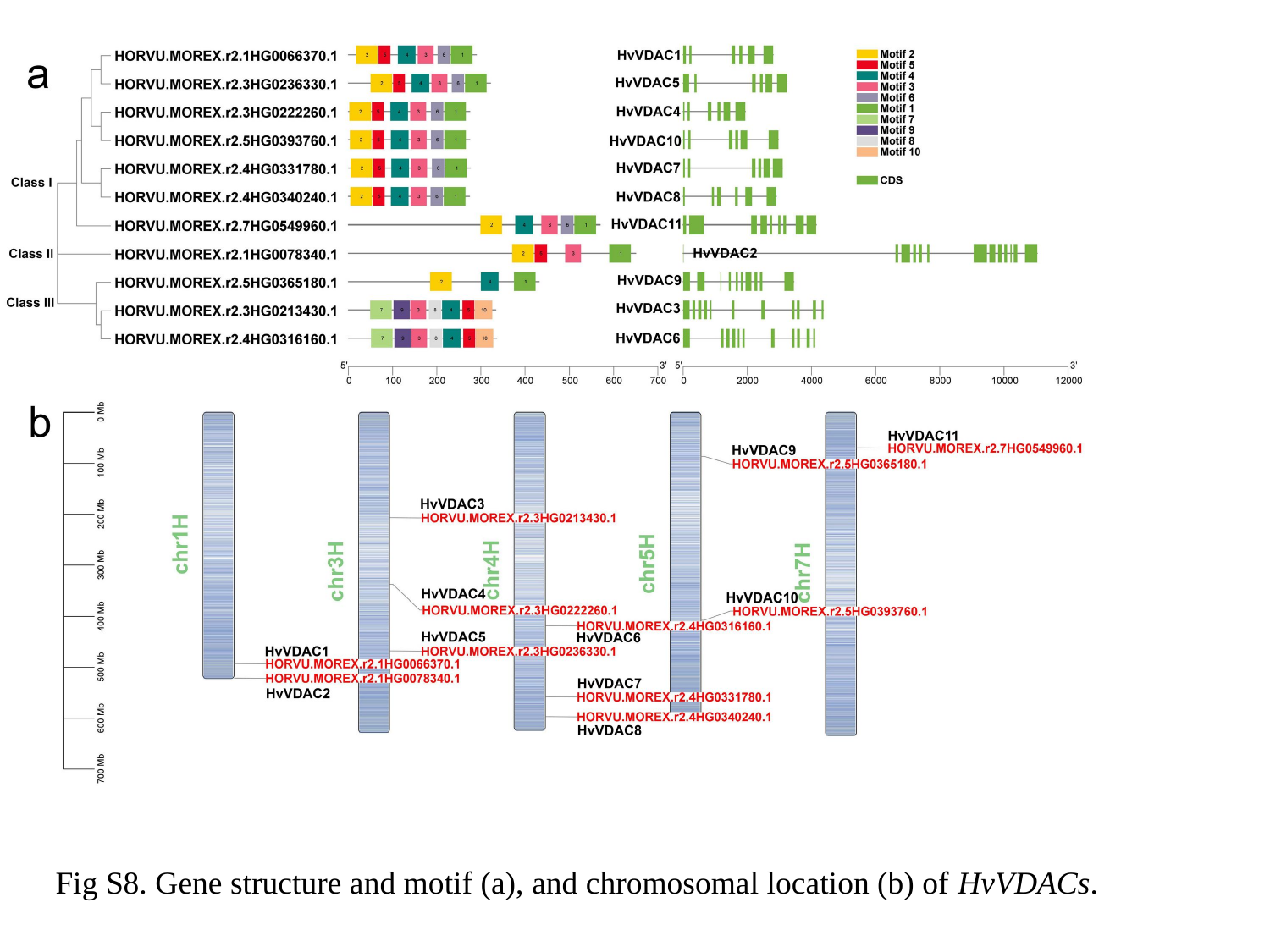

Fig S8. Gene structure and motif (a), and chromosomal location (b) of HvVDACs.

## Slide 9
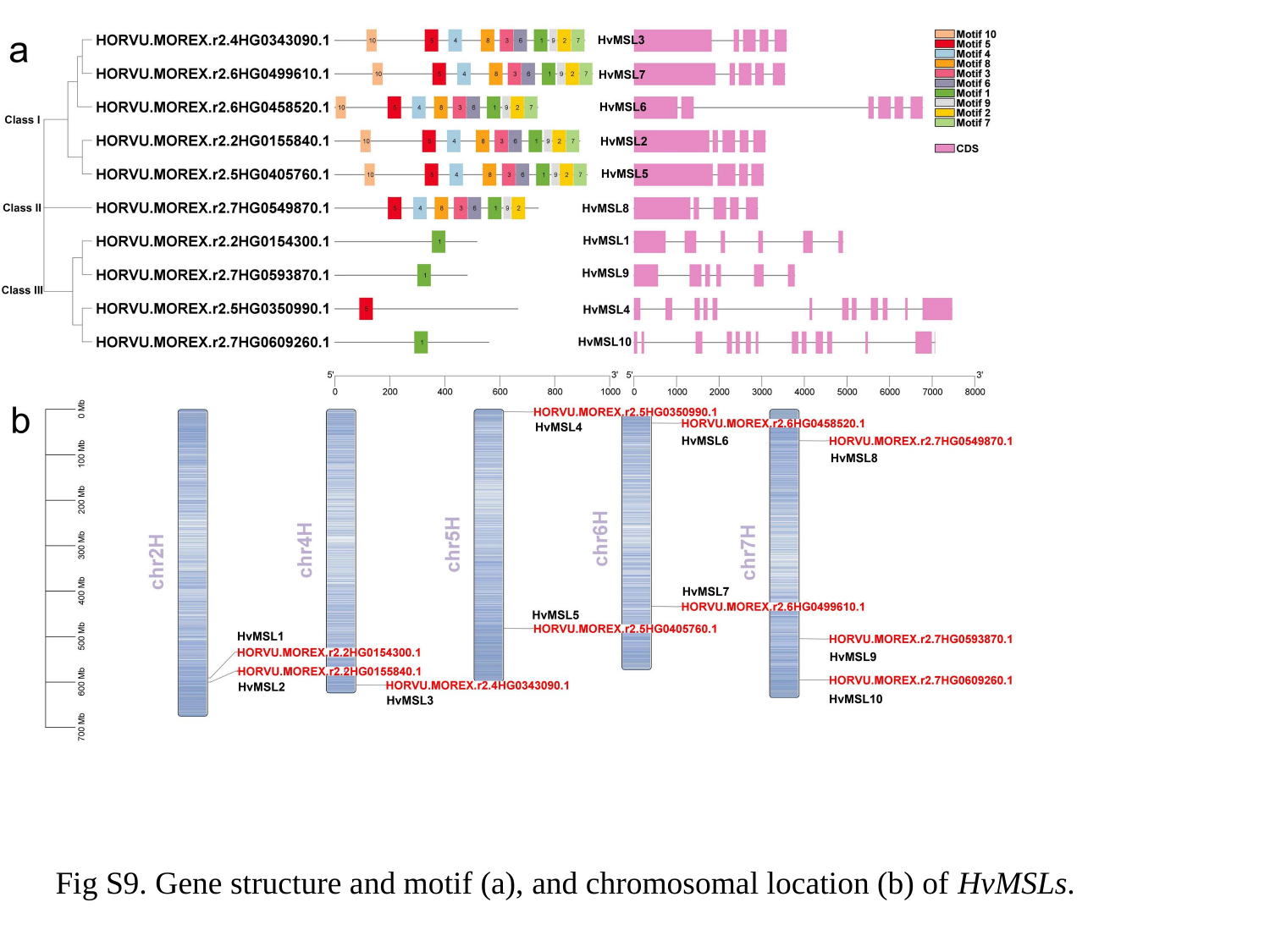

Fig S9. Gene structure and motif (a), and chromosomal location (b) of HvMSLs.
